# Inflammatory and Immune Cytoprofiles of Active Ulcerative Colitis from Crohn’s Disease—Insights from Multivariable Modeling

**DOI:** 10.3390/ijms27167217

**Published:** 2026-08-13

**Authors:** Małgorzata Krzystek-Korpacka, Łukasz Lewandowski, Iwona Bednarz-Misa, Andrzej Korpacki, Katarzyna Neubauer

**Affiliations:** 1 Department of Biochemistry and Immunochemistry, Faculty of Medicine, Wroclaw Medical University, 50-556 Wroclaw, Poland; lukasz.lewandowski@umw.edu.pl (Ł.L.); iwona.bednarz-misa@umw.edu.pl (I.B.-M.); 2 Department of Gastroenterology, Hepatology and Internal Medicine, Faculty of Medicine, Wroclaw Medical University, 50-556 Wroclaw, Poland; andrzej.korpacki@student.umw.edu.pl (A.K.); katarzyna.neubauer@umw.edu.pl (K.N.)

**Keywords:** cytokine, inflammatory bowel disease, fibrosis, eosinophil–mast cell axis, GM-CSF, TRAIL, CTACK, MIP-1β (CCL4), SCF, SDF-1α

## Abstract

Differentiating active ulcerative colitis (UC) from Crohn’s disease (CD) is one of the unmet needs addressed by biomarkers in inflammatory bowel disease (IBD). The immune landscapes of UC and CD differ, justifying the search for discriminatory markers and novel therapy targets among their mediators. Herein, 27 systemic cytokines were measured using flow cytometry-based methodology in 138 IBD patients, with an additional 21 being determined in 67 of the patients. Their discriminatory power was assessed individually and as exploratory multivariable signatures generated using logistic regression, hierarchical clustering, and principal component analysis. Eotaxin-1, macrophage inflammatory protein (MIP)-1β, and tumor necrosis factor-related apoptosis-inducing ligand (TRAIL) showed fair discriminatory potential, while multivariable models performed better. Interleukin (IL)-1β, IL-4, and MIP-1α were strongly associated with CD, whereas IL-5, granulocyte-macrophage colony-stimulating factor (GM-CSF), MIP-1β, and TRAIL were associated with UC. Active UC was characterized by mediators linked to eosinophil-, mastocyte-, and neutrophil-driven inflammation and tissue repair (eotaxin-1, IL-5, growth-regulated oncogene (GRO), MIP-1β, stem cell factor (SCF), GM-CSF, IL-1 receptor antagonist, TRAIL, stem cell growth factor (SCGF)-β, and cutaneous T cell-attracting chemokine (CTACK)), whereas active CD was associated with Th1/Th17 immunity, myeloid activation, fibrosis, angiogenesis, and neuroimmune remodeling (IL-1β, IL-12p70, IL-15, ‘regulated on activation, normal T-cell expressed and secreted’ (RANTES), MIP-1α, stromal cell-derived factor (SDF)-1α, nerve growth factor β (β-NGF), and leukemia inhibitory factor (LIF)). In conclusion, integrated circulating immune signatures identify several understudied cytokines as potential contributors to disease-specific pathways and show potential in distinguishing active UC from CD warranting further mechanistic studies and independent validation in larger cohorts.

## 1. Introduction

Inflammatory bowel disease (IBD) encompasses two major clinical entities, ulcerative colitis (UC) and Crohn’s disease (CD), both currently incurable and characterized by chronic, relapsing inflammation of the gastrointestinal tract. Although their precise etiology remains incompletely understood, current evidence indicates that IBD results from an inappropriate immune response to intestinal microbiota in genetically predisposed individuals exposed to environmental triggers. The resulting disruption of intestinal homeostasis leads to persistent activation of innate and adaptive immune pathways, ultimately causing tissue damage and impaired mucosal healing [1,2,3]. Historically a “Western disease”, IBD is becoming a global health care problem with rising incidence and falling mortality, leading to a phenomenon referred to as “compounding prevalence” [4]. In Poland, data from the National Health Fund has shown that the CD and UC prevalence was respectively 61.6 and 191.4 per 100,000. In turn, the incidence of CD was 4.7 per 100,000, and that of UC 12.5. Moreover, a substantial increase in the disease burden over the years 2009–2020 was demonstrated [5].

Active CD and UC both manifest with diarrhea, abdominal pain, fatigue, weight loss, rectal bleeding, urgency, and impaired quality of life. Systemic symptoms such as anemia, fever, malnutrition, and extraintestinal involvement may occur in both conditions, particularly in patients with moderate-to-severe disease activity. Their intensity often reflects the degree of mucosal inflammation rather than the specific IBD subtype, leading to considerable clinical overlap. Consequently, symptom-based assessment alone has limited discriminatory value, especially in individuals presenting with colonic CD, which may closely resemble UC, or in newly diagnosed patients, in whom certain manifestations traditionally associated with one disease phenotype—such as transmural complications and fistula formation in CD or continuous rectal inflammation in UC—may not always be evident at disease onset. This clinical overlap not only complicates the initial diagnostic workup but may also delay the implementation of disease-specific therapeutic strategies and/or expose patients to ineffective or unnecessary treatments, avoidable adverse events, or inappropriate surgical decision-making [1,2,3,6,7,8].

Diagnostic uncertainty at disease onset remains a challenge, leaving ca. 5–15% of adult patients and even more children without a specific diagnosis (referred to as IBD-unclassified; IBDU) or necessitating reclassification during follow-up, mostly IBDU or UC into CD [6,7,8,9]. Definitive diagnosis frequently requires ileocolonoscopy, histopathological examination, and cross-sectional imaging. However, these approaches are invasive, costly, and unsuitable for repeated use in routine monitoring, creating a strong need for non-invasive alternatives that could assist in both diagnosis and disease classification [10,11,12]. The ability to distinguish active UC from CD is regarded as one of the five core clinical purposes of biomarkers in IBD [13]. Among currently available non-invasive markers, C-reactive protein (CRP) and fecal calprotectin are the most extensively validated. These biomarkers correlate reasonably well with inflammatory activity and can help differentiate intestinal inflammation from functional gastrointestinal disorders. However, despite their utility in identifying active IBD, they generally lack sufficient specificity to reliably distinguish CD from UC [10,12,13,14]. Moreover, these and other currently available biomarkers lack sufficient power to serve as treatment targets, necessitating further search for non-invasive measures serving both purposes [15,16,17].

Cytokines, chemokines, and growth factors are key regulators of inflammatory and immune responses and major determinants of disease phenotype, severity, and progression. Accumulating evidence over the past three decades has shown that cytokine networks are not merely byproducts of inflammation but active drivers of IBD pathogenesis. This is underscored by the clinical success of biologic therapies targeting tumor necrosis factor (TNF)-α, interleukin (IL)-12/23, and related immune pathways [18,19,20]. Although UC and CD share several pathogenic mechanisms, they differ in their predominant immune profiles [21]. Crohn’s disease has classically been associated with Th1/Th17 responses, characterized by increased interferon (IFN)-γ, TNF-α, IL-12, IL-17, and IL-23, whereas UC has been linked to an atypical Th2-like response involving IL-5 and IL-13. However, current evidence indicates substantial overlap between cytokine networks as well as marked inter-patient heterogeneity. Despite this overlap, differences in the magnitude and composition of inflammatory signaling contribute to the distinct pathological features of UC and CD, including mucosal inflammation in UC and transmural, often fibrostenotic, inflammation in CD [18,19,22,23,24]. Consequently, circulating inflammatory and immune mediators may be promising minimally invasive biomarkers reflecting disease-specific immunological activity.

While individual biomarkers frequently display limited diagnostic specificity, emerging evidence suggests that composite immune signatures generated from multiple circulating mediators may improve the differentiation of UC and CD. Multiplex profiling approaches might help in identifying distinct combinations of cytokines, chemokines, and growth factors associated with each phenotype, supporting the concept that systemic immune fingerprints can complement conventional diagnostic methods. Furthermore, such biomarker panels may provide insights into disease behavior, therapeutic responsiveness, and prognosis, thereby contributing to the development of personalized management strategies in IBD [25,26,27]. It is of utmost clinical relevance as current strategies fall short of expectations. This is clearly demonstrated by findings of a large multicentre cross-sectional study, which have unequivocally shown that suboptimal disease control affects as many as 52.2% of CD and 44.3% of UC patients, predominantly due to impaired quality of life, clinically significant extraintestinal manifestations, steroid overuse, signs of active inflammation in UC and CD, and active fistulas in CD [28]. The huge breakthrough that followed the introduction of biologics raised hopes of a complete reversal of the natural history of IBD. It has, however, become apparent that not all patients respond to these new therapies, and some, despite an initial good response, lose it as treatment continues. This set of phenomena has been termed the ‘therapeutic ceiling’. Gaining a better understanding of the disease pathogenesis with further research into drugs, potentially targeting broader networks of cytokines and immune cell signalling pathways rather than individual cytokines, has, in turn, been proposed as a measure to break through [29,30,31].

Therefore, the aim of this study was to evaluate a broad panel of systemic inflammatory and immune mediators as potential discriminatory markers of active UC versus CD, both individually and as components of exploratory multivariable signatures generated using multivariable modeling techniques, including logistic regression, hierarchical clustering, and principal component analysis (PCA) in a relatively large cohort of patients with confirmed diagnosis of UC or CD not subjected to biological therapies.

## 2. Results

Multiplex assays were used to determine the concentration of various immunomodulatory mediators: IL-1β, IL-1 receptor antagonist (RA), IL-4, IL-5, IL-6, IL-7, IL-8, IL-9, IL-10, IL-12p70, IL-13, IL-15, IL-17, eotaxin-1, fibroblast growth factor (FGF)-2, granulocyte colony-stimulating factor (G-CSF), granulocyte-macrophage colony-stimulating factor (GM-CSF), IFNγ, IFNγ-induced protein 10 kDa (IP10), monocyte chemoattractant protein (MCP-1), macrophage inflammatory protein (MIP)-1α, MIP-1β, TNFα, vascular endothelial growth factor (VEGF)-A, platelet-derived growth factor (PDGF)-BB, ‘regulated upon activation, normal T-cell expressed, and secreted’ (RANTES) in 138 patients with active CD or UC (dataset A). Additionally, in a subgroup of 67 patients (dataset B), IL-2RA, IL-3, IL-12p40, IL-16, IL-18, cutaneous T-cell-attracting chemokine (CTACK), growth-regulated oncogene (GRO), hepatocyte growth factor (HGF), IFNα, leukemia inhibitory factor (LIF), MCP-3, macrophage colony-stimulating factor (M-CSF), macrophage migration inhibitory factor (MIF), monokine induced by gamma interferon (MIG), nerve growth factor β (β-NGF), granulocyte colony-stimulating factor (CSF), stem cell growth factor (SCGF)-β, stromal cell-derived factor (SDF)-1α, and TNF-related apoptosis-inducing ligand (TRAIL) were measured. Firstly, groupwise univariate comparisons in each separate immunomodulator between these groups (active CD vs. UC) were conducted and their discriminatory power assessed using receiver operating characteristics (ROC) analysis. Secondly, multivariable logistic regression models with several feature selection strategies (based on stepwise regression) were developed in the whole population sample and the mentioned subset of 67 patients to analyze the multivariable association of inflammatory and immune mediators and the odds of suffering from UC. Due to multicollinearity between some analytes (determined based on Pearson’s r > 0.80), hierarchical clustering and subsequent PCA within each cluster were utilized, generating principal components (PC), which were then mixed with the other mediators for which the magnitude of correlation did not meet the mentioned criterion. To characterize internal discrimination of the multivariable models, area under ROC curves (AUC) was estimated using 10-fold cross-validation.

### 2.1. Univariate Differences in the Concentration of Inflammatory and Immune Mediators in Context of Active CD and UC

Among all evaluated inflammatory and immune mediators, eight were statistically significantly different between the groups (Table 1). Active UC was characterized by higher median concentrations of: eotaxin-1 (by 47.9%), MIP-1β (by 24.2%), MCP-1 (by 61.6%), TRAIL (by 19.1%), CTACK (by 28.2%), VEGF-A (by 62.3%), SCF (by 23%), and SCGF-β (by 26.2%). The differences in concentrations of M-CSF and IFNγ were on the verge of statistical significance.

### 2.2. Discriminatory Power of Individual Inflammatory and Immune Mediators in Differentiating Between Active CD and UC

**Figure 1:**
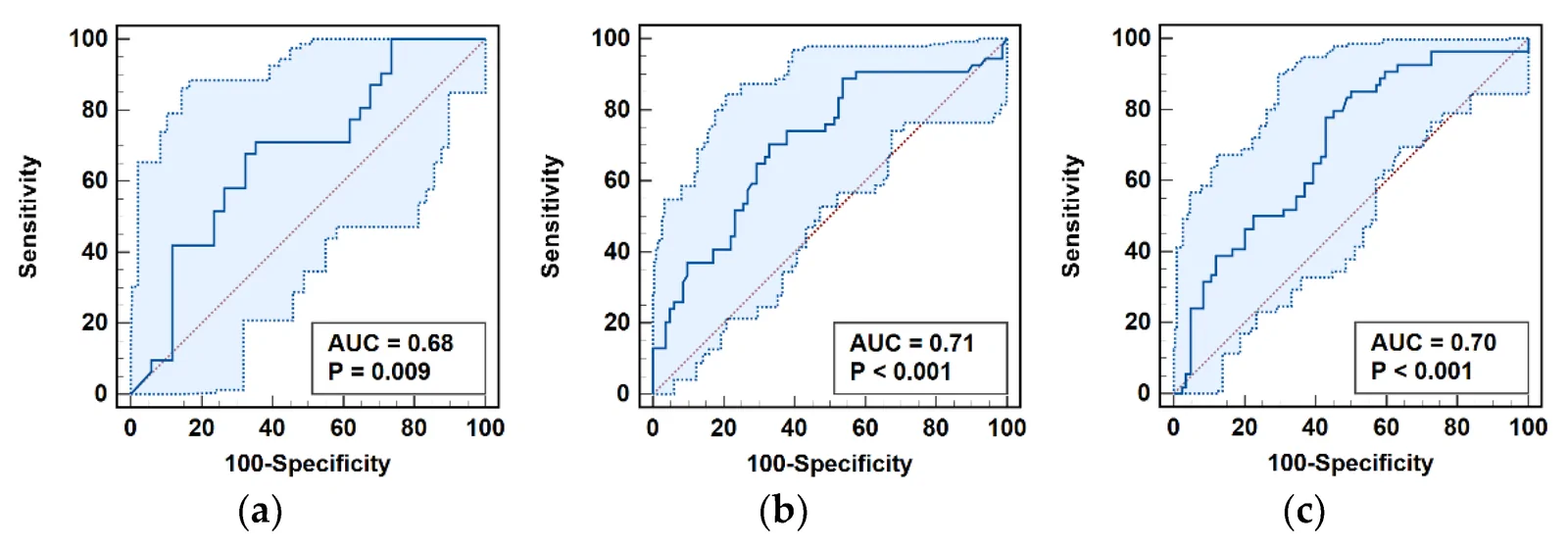

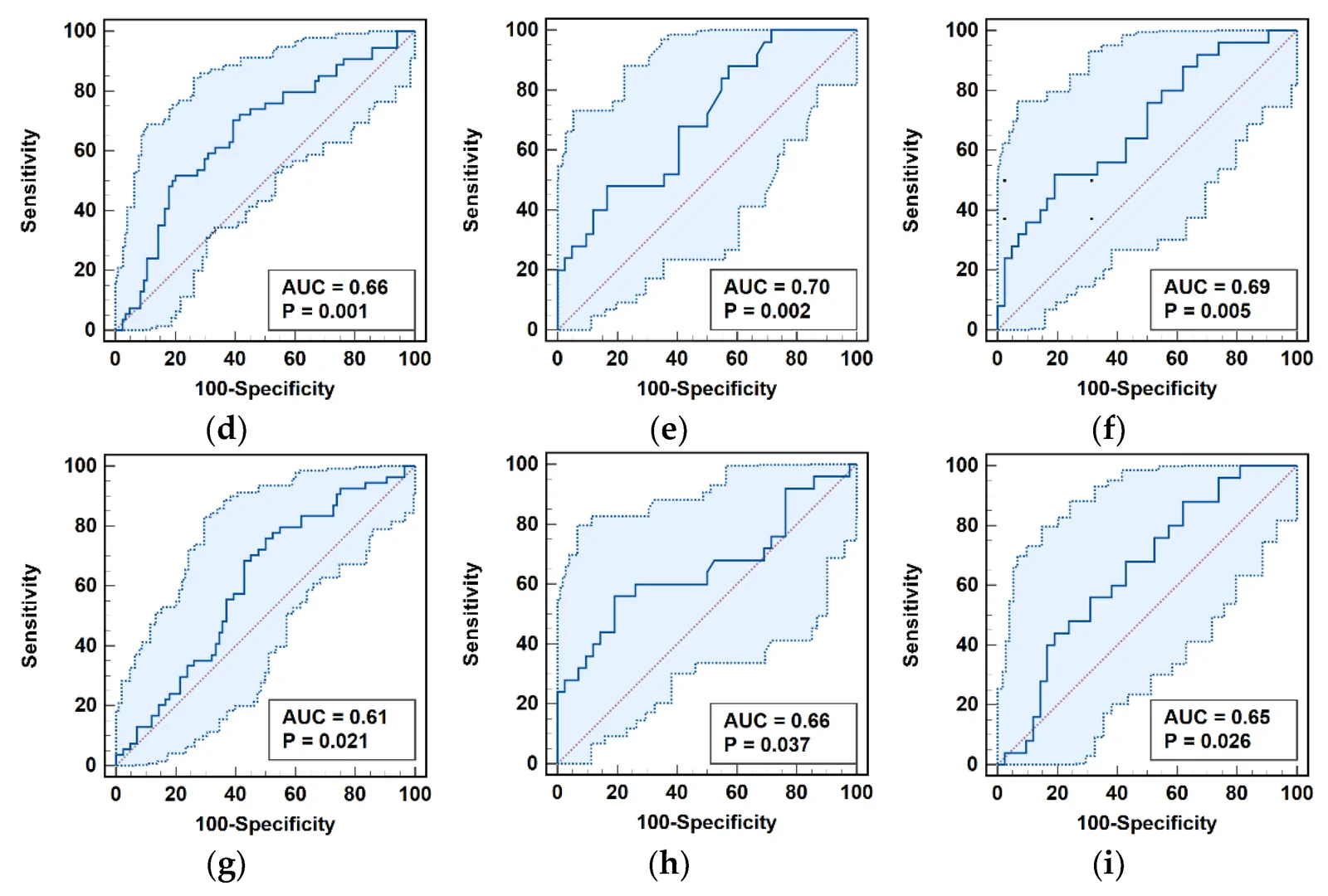
Receiver operating characteristics (ROC) curves for selected cytokines, chemokines, and growth factors as potential discriminatory markers for active UC: (**a**) CRP; (**b**) eotaxin-1; (**c**) MIP-1β; (**d**) MCP-1; (**e**) TRAIL; (**f**) CTACK; (**g**) VEGF-A; (**h**) SCF; (**i**) SCGFβ. AUC, area under receiver operating characteristics (ROC) curve; CRP, C-reactive protein; MIP-1β, macrophage inflammatory protein 1β; MCP-1, monocyte chemoattractant protein 1; TRAIL, tumor necrosis factor-related apoptosis-inducing ligand; CTACK, cutaneous T-cell-attracting chemokine; VEGF-A, vascular endothelial growth factor A; SCF, stem cell factor; SCGFβ, stem cell growth factor-β. ROC curve is indicated by solid blue line, 95% confidence interval is marked by dashed blue lines, and the performance of a marker without any discriminatory power (AUC = 0.5) is indicated by a diagonal red line.

Inflammatory and immune mediators found significantly different between active UC and CD in univariate analysis were evaluated using ROC analysis as potential differential markers and their performance was compared to that of CRP. Their overall accuracy was quite similar, ranging from poor to fair (Figure 1, Table 2).

The highest AUC was obtained for eotaxin-1 (fair accuracy), followed by TRAIL (fair accuracy), MIP-1β (fair accuracy), and CTACK (poor accuracy), all superior to CRP (poor accuracy). Eotaxin-1 also had the highest, although low, Youden index, indicative of the best combined sensitivity and specificity associated with the optimal cut-off value (Table 2).

### 2.3. The Multivariable Association Between Chemokines, Cytokines, and Growth Factors and the Odds of UC in Context of Differentiation Between Active CD and UC

**Figure 2:**
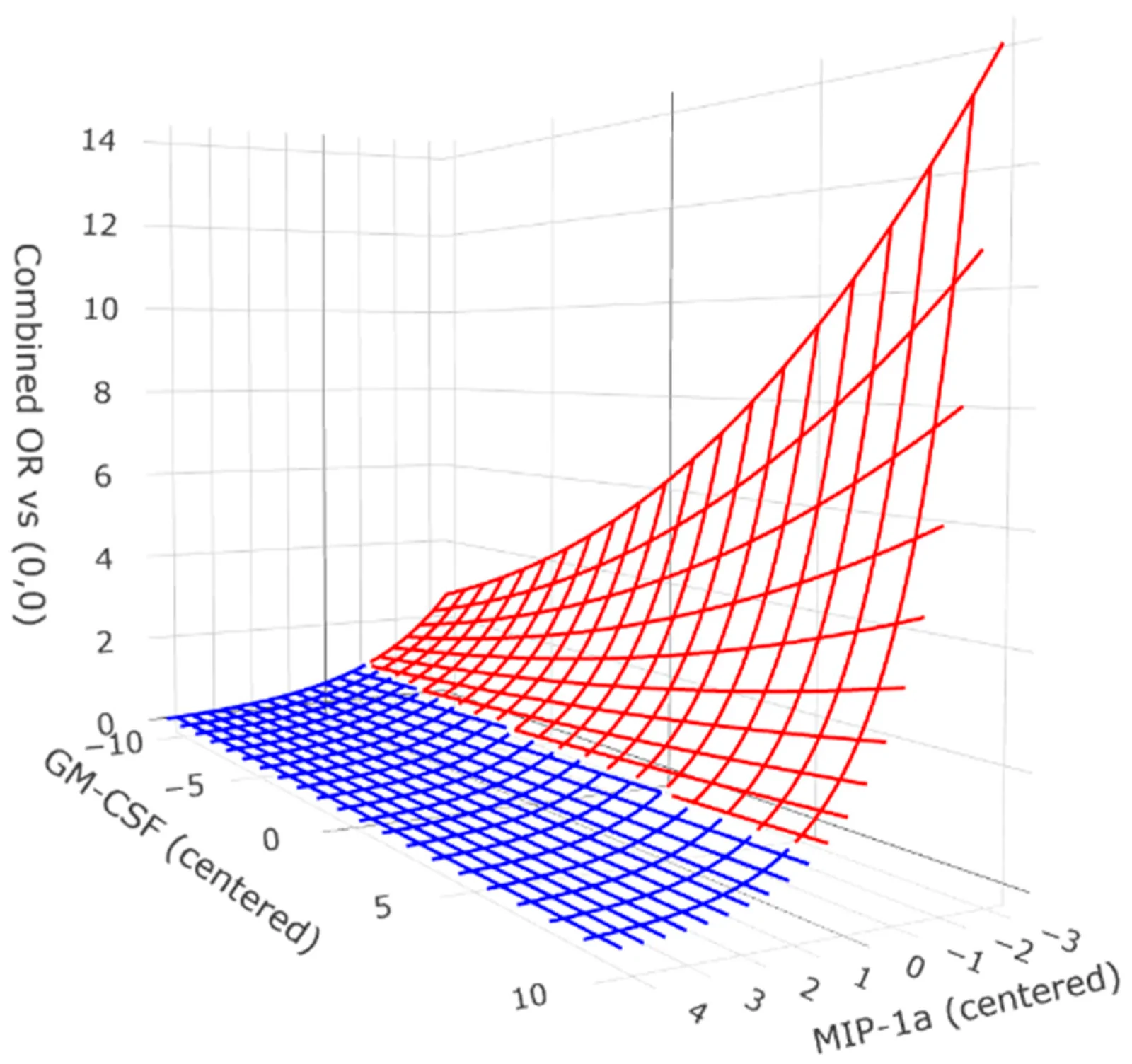
The change in odds associated with interaction between GM-CSF and MIP-1α, based on model 3. GM-CSF, granulocyte-macrophage colony-stimulating factor; MIP-1α, macrophage inflammatory protein α.

The multivariable model presented in this section was the result of feature selection; the intermediate models (1 and 2) are shown in the Supplementary Materials. The feature selection was aimed at choosing which inflammatory/immune mediators were associated with higher odds of UC among patients with active IBD (UC or CD). According to the chosen multivariable model yielded through one modeling strategy (model 3, shown in Table 3), the following cytokines were statistically significantly associated with higher odds of UC: IL1-RA (by 1.3% per each one-unit increase in concentration), IL-5 (by 1.5-fold per concentration unit), and IFNγ (by 3.6%). The odds of UC were negatively associated with cytokines: IL-1β (5-fold increase in CD odds per concentration unit), IL-4 (1.7-fold increase in CD odds per concentration unit), and IL-10 (increase in CD odds by 7.2% per concentration unit). Moreover, UC odds were positively associated with chemokines: eotaxin-1 (by 2.1%) and MIP-1β (by 5.4%) and—negatively—with RANTES (increased CD odds by 1.9%). MIP-1α and GM-CSF interacted with each other in their modulation of the odds. The negative association between the odds and MIP-1α started to statistically significantly increase its magnitude with increasing GM-CSF between the 3rd and 4th decile of GM-CSF. Conversely, the positive association between the odds and GM-CSF weakened with increasing MIP-1α, becoming insignificant between the 6th and 7th deciles of MIP-1α (Figure 2).

According to the multivariable model containing fewer records but a larger initial number of candidate variables used for feature selection (model 4, shown in Table 4), the odds of active UC were positively associated with IL-1RA (by 1.6% per unit increase in concentration), TRAIL (by 7.6%), GRO (by 3.1%), GM-CSF (by 9.9%), SCF (by 3.4%), and MIP-1β (by 5.1%). In addition, PC1 was retained as an aggregate mediator profile derived from PCA of highly correlated cytokines, chemokines, and growth factors. Each one-unit increase in PC1 was associated with a 9.09-fold increase in the odds of active UC (*p* = 0.002). Because all PC1 eigenvectors were negative in the present PCA orientation, this component should be interpreted as a latent multivariable axis rather than as a simple increase in all contributing mediators. To aid interpretation, the approximate back-projected contributions of the individual standardized mediators to the PC1-associated log-odds direction are shown in Table 5. These values should not be interpreted as independent single-marker odds ratios.

**Figure 3:**
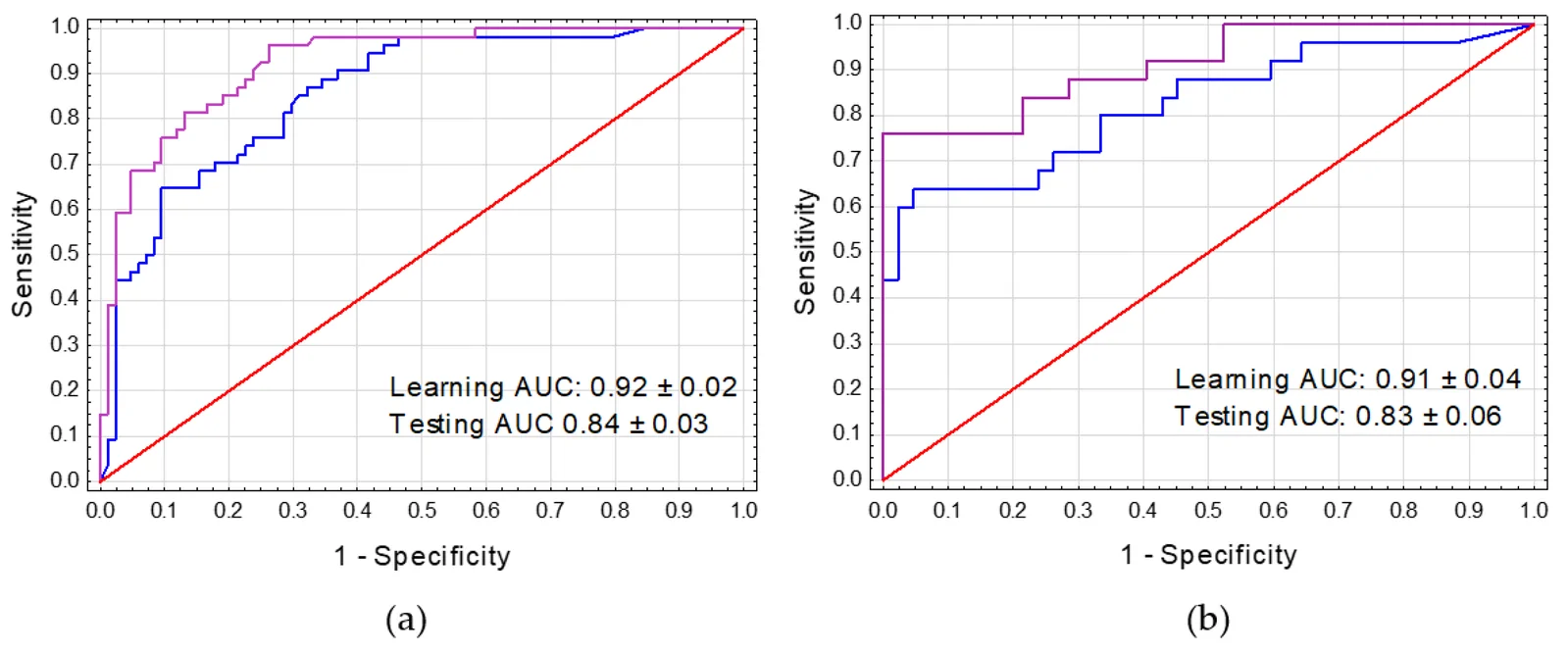
Learning and testing receiver operating characteristics (ROC) curves for multivariable logistic regression models: (**a**) model 3 trained on dataset A; (**b**) model 4 trained on dataset B. Learning ROCs are shown in magenta, while testing ROCs in blue. The performance of a marker without any discriminatory power (AUC = 0.5) is indicated by a diagonal red line. AUC, area under receiver operating characteristics (ROC) curve.

Both models were subjected to 10-fold cross-validation after the model structures had been selected. This procedure provided an internal post-selection assessment of discrimination. The model shown in Table 3 yielded a testing AUC of 0.841 ± 0.033, with testing sensitivity of 74.1% and testing specificity of 79.8% at the threshold maximizing Youden’s index. The model shown in Table 4 yielded a learning AUC of 0.91 ± 0.037 and a testing AUC of 0.83 ± 0.056. At the probability threshold maximizing Youden’s index in the testing subset (J = 0.592), sensitivity was 64.0% and specificity was 95.2%. Thus, the revised extended-panel model showed high specificity but only moderate sensitivity in the testing subset. Examples of testing ROCs for both models are shown in Figure 3a and Figure 3b, respectively.

## 3. Discussion

The ability to distinguish active UC from CD represents one of the five major unmet needs that biomarkers are expected to address in IBD [13]. Although numerous circulating inflammatory and immune mediators have been reported to be dysregulated in IBD [26,27,32,33,34,35,36,37,38], including an assessment of the association between genetically determined levels of circulating cytokines and the risk of developing IBD using the Mendelian randomization method [39], relatively few studies have evaluated their utility in differentiating active UC from CD [26,33,38,40]. Moreover, most investigations have been limited either by small patient cohorts or by focusing on a restricted panel of well-established cytokines, potentially overlooking novel biomarkers and therapeutic targets.

Using high-throughput cytokine profiling in a relatively large IBD cohort, we identified several mediators with fair individual discriminatory value, including eotaxin-1, MIP-1β, and TRAIL, and developed exploratory multivariable signatures showing greater internal discrimination. More importantly, these analyses highlighted less extensively studied mediators such as SCF, SCGFβ, CTACK, TRAIL, MIP-1β, GRO, LIF, β-NGF, and SDF-1α as components of distinct inflammatory and immune signatures associated with UC and CD, thereby providing insights into disease-specific immunopathology and identifying potential targets for future mechanistic and therapeutic studies. Notably, the multivariable modeling strategy applied here evaluates mediator effects within the context of an interconnected inflammatory network, providing a biologically more realistic representation of IBD pathogenesis than analyses based on isolated biomarkers. Overall, the identified signatures suggest that active UC is characterized primarily by eosinophil-, mast cell-, and neutrophil-associated mucosal inflammation accompanied by tissue repair responses, whereas active CD is more strongly associated with Th1/Th17-driven immunity, myeloid activation, fibrosis, angiogenesis, and neuroimmune remodeling.

A prominent biological theme emerging from our analyses was the association of active UC with mediators linked to type-2 immunity, the eosinophil–mast cell axis, and neutrophil-derived mucosal inflammation. UC has traditionally been associated with enhanced Th2-like responses, increased innate lymphoid cells (ILC) group 2 activity, and greater eosinophil involvement in intestinal inflammation [41,42]. Consistent with this paradigm, eotaxin-1 was among the strongest individual discriminators between UC and CD and remained an independent contributor to model 3, where each one-unit increase in concentration increased the odds of UC by 2%. As a principal eosinophil chemoattractant, eotaxin-1 promotes eosinophil recruitment and activation, and its expression correlates with eosinophil infiltration and disease severity in UC [33,43]. While IL-13, another cytokine frequently linked to UC [32,33], did not demonstrate discriminatory value in our cohort, IL-5 showed a significant positive association with the odds of UC, with an approximately 1.5-fold increase in the odds per unit increase. Like eotaxin-1, IL-5 is closely associated with Th2 cells, ILC2s, and eosinophils [18,40,44] and contributes to epithelial injury and chronic mucosal inflammation through eosinophil activation and survival [44,45].

These findings also point toward a broader role of mast cell–eosinophil interactions in UC, especially important considering growing recognition of mast cell activation syndrome relevance to the pathogenesis of chronic inflammatory diseases [46]. Both eotaxin-1 and IL-5 are produced by mast cells and participate in a self-amplifying mast cell–eosinophil inflammatory circuit that is increasingly recognized as an important component of IBD pathophysiology, particularly in UC [47,48,49,50]. In this context, the positive associations of SCF, MCP-1 (CCL2), and GM-CSF with UC observed here are biologically plausible. Eosinophil-secreted SCF is essential for mast cell development, survival, and activation, whereas MCP-1 and GM-CSF can be released by mast cells and modulate eosinophil recruitment and function [47,51]. The observed substantial positive effects of SCF and GM-CSF in model 4 (odds increase per concentration unit by 3.4 and 9.9%, respectively), together with the individual discriminatory performance of SCF and MCP-1, therefore suggest that enhanced mast cell activity represents an additional feature distinguishing active UC from CD. Collectively, these results support the concept that eosinophil-rich inflammation, sustained by mast cell–eosinophil cross-talk and type-2-associated immune pathways, constitutes an important component of the systemic inflammatory signature of active UC.

In addition, we found active UC to be associated with mediators reflecting neutrophil-driven mucosal inflammation and accompanied by compensatory immune-regulatory and tissue repair responses. GRO (CXCL1), a potent neutrophil chemoattractant, was positively associated with UC, increasing its odds by 3.1% with each one-unit increase in its concentration. This finding is consistent with the predominantly superficial, neutrophil-rich inflammation characteristic of UC, including cryptitis and crypt abscess formation. Recent studies have identified CXCL1 as a marker of UC activity that correlates with neutrophil and dendritic-cell infiltration and decreases with clinical remission [52,53,54,55]. Together with the eosinophil-associated signals discussed above, the positive association of GRO further supports the concept that active UC is characterized by prominent recruitment of innate immune cells to the colonic mucosa.

Notably, several mediators positively associated with UC are better known for their anti-inflammatory, homeostatic, or reparative functions than for directly promoting inflammation. Among these, IL-1 receptor antagonist (IL-1RA) showed a consistent positive association with UC across both models. As a natural inhibitor of IL-1 signaling, IL-1RA counteracts the pro-inflammatory effects of IL-1α and IL-1β and exerts protective effects in experimental intestinal inflammation [56]. Although previous studies have reported conflicting findings regarding its relative concentrations in UC and CD [36,57], its positive association with UC in our study may indicate a stronger compensatory attempt to limit mucosal inflammatory injury. Similarly, GM-CSF, which contributed positively to UC discrimination, increasing UC odds by 6.6% and 9.9% per unit increase in models 3 and 4, respectively, may reflect both enhanced mucosal repair and preservation of innate immune homeostasis. This interpretation is consistent with evidence that impaired GM-CSF signaling and neutralizing anti-GM-CSF autoantibodies are more characteristic of CD [58,59,60].

TRAIL (TNFSF10) further supports the notion that regulatory and restorative pathways contribute to the UC-associated signature. Its concentrations were significantly higher in UC and it performed well both individually and as an independent component in model 4, increasing UC odds by 7.6% per each one-unit increase in its concentration. Although primarily recognized as an apoptosis-inducing member of the TNF superfamily, TRAIL also exerts important immunomodulatory functions. Experimental studies have demonstrated that TRAIL suppresses colitis by inhibiting activation of colitogenic T cells and attenuating intestinal inflammation [61], while broader evidence supports a role in maintaining immune tolerance and limiting autoimmune responses [62,63]. Elevated TRAIL concentrations may therefore reflect activation of endogenous mechanisms promoting resolution of inflammation and mucosal healing, a therapeutic objective that has become central to UC management [64,65].

Interestingly, MIP-1β (CCL4) emerged as one of the most robust UC-associated mediators, demonstrating both strong individual discriminatory performance and consistent inclusion in the multivariable models, where it increased UC odds by over 5% per concentration unit. This observation agrees with recent evidence identifying MIP-1β as a circulating protein causally associated with increased susceptibility to UC but not CD [66]. All macrophage inflammatory proteins (MIPs) are involved in intestinal inflammation and neoplastic transformation [67,68]. Functionally, MIP-1β recruits monocytes/macrophages, activated T cells, and NK cells and thereby contributes to the maintenance of intestinal inflammation [69]. However, its divergent behavior from MIP-1α (CCL3) in our models suggests that these closely related chemokines may characterize distinct immune environments, with MIP-1β being more reflective of the UC-associated inflammatory profile.

In addition to GM-CSF, other less-studied mediators positively associated with UC appeared to reflect tissue maintenance and repair. GM-CSF, SCGFβ (CLEC11A) and M-CSF are all involved in hematopoietic progenitor-cell maintenance and myeloid-cell development [70,71]. SCGFβ is said to be a systemic marker of enhanced cellular renewal and tissue repair responses accompanying chronic mucosal injury [70]. Accordingly, its silencing in gastric cancer has downregulated immunosuppressive and healing-promoting M2 macrophages, Tregs, and myeloid-derived suppressor cells while upregulating proinflammatory CD8+ T cells [72]. Likewise, M-CSF promotes M2 polarization [71]. CTACK (CCL27), in turn, regulates trafficking of epithelial-associated lymphocytes [73] and may indicate enhanced barrier-oriented immune surveillance, although direct evidence for the intestine is lacking. Taken together, the positive contributions of GRO, IL-1ra, GM-CSF, TRAIL, MIP-1β, SCGFβ, and CTACK suggest that the systemic immune signature of active UC reflects not only ongoing mucosal inflammation, but also simultaneous activation of counter-regulatory and tissue repair mechanisms aimed at restoring epithelial integrity and immune homeostasis.

A second major theme emerging from our analyses was the association of active CD with mediators linked to Th1/Th17 immunity, myeloid activation, granulomatous inflammation, and chronic inflammatory responses, the latter known to be stronger at systemic level in CD than UC [12,15,74,75]. This pattern was most clearly reflected by the strong negative association of IL-1β with the odds of UC, whereby increasing IL-1β concentrations increased the odds of CD by over five-fold per concentration unit. IL-1β is a central pro-inflammatory cytokine that promotes leukocyte recruitment, cytokine production, epithelial barrier disruption, and differentiation of Th17 cells and ILC3s, all hallmarks of the immune environment characteristic of CD [23,74,76]. Consistent with these findings, Górecka et al. reported higher circulating concentrations of IL-1α and IL-12p70 in active CD compared with UC [40]. Although IL-1α could not be reliably quantified in our study, IL-12p70 contributed negatively to UC discrimination through PC1, supporting the notion of a stronger Th1-polarized immune response in CD. Likewise, IL-15, another component of PC1, is known to promote ILC3, Th1, and Th17 responses while sustaining leukocyte activation and memory T-cell survival, thereby contributing to disease chronicity and relapse [23,77,78]. Notably, inhibition of IL-15/IL-15R signaling represents one of the mechanisms through which infliximab exerts anti-inflammatory effects in IBD [79].

The predominance of Th1/Th17-driven inflammation in CD may also explain the inverse associations observed for several regulatory cytokines. Although IL-4 is traditionally associated with Th2 and ILC2 responses [41], higher circulating concentrations were associated with the odds of CD, particularly in model 3, where an increase in one unit of interleukin concentration increased CD odds by 1.7-fold. Because systemic cytokine concentrations do not necessarily reflect mucosal activity, elevated IL-4 may represent a compensatory anti-inflammatory response to the greater systemic inflammatory burden observed in CD [12,15,74], as evidenced by the higher CRP concentrations in our cohort. Alternatively, it may reflect activation of regulatory pathways aimed at counterbalancing dominant Th1/Th17 immunity [18,19,22]. A similar interpretation may apply to IL-10, which was also associated with increased CD odds despite its well-established anti-inflammatory properties and ability to suppress Th1/Th17 responses [19,22,24]. Although reports on circulating IL-10 concentrations in IBD have been inconsistent [26,34,37,80,81], Mendelian randomization analyses have recently identified IL-10 among circulating proteins causally associated with increased CD risk [66]. Together, these observations suggest that elevated systemic levels of anti-inflammatory mediators may not necessarily indicate reduced inflammation but may instead reflect compensatory regulatory responses to a stronger underlying inflammatory drive. However, IL-10 association with CD may also reflect the presence of “creeping fat”, a CD hallmark and a rich source of the interleukin [82] or be a consequence of its underestimation in UC due to the presence of autoantibodies. Although detected in both UC and CD, autoantibodies against IL-10 are associated with major histocompatibility complex (HLA)-DRB1*01:03, the strongest HLA susceptibility allele for UC and one particularly prevalent in individuals of European ancestry [83,84].

Additional support for a CD-associated myeloid and Th1/Th17 inflammatory signature was provided by RANTES (CCL5) and MIP-1α, both of which were negatively associated with the odds of UC. RANTES is highly expressed within granulomas, a histopathological hallmark of CD, and recruits C-C motif chemokine receptor (CCR) 5- and CCR3-expressing T cells, including Th1-polarized and memory CD4+ lymphocytes involved in granulomatous inflammation [85,86]. It also promotes recruitment of pathogenic CCR5+ Th17 cells and fibrocytes implicated in intestinal fibrosis [34,87]. Elevated numbers of circulating fibrocytes are observed in both CD and UC but increased concentrations of their chemoattractants, RANTES and SDF-1α (CXCL12), have been reported only in CD [34]. Similarly, MIP-1α is closely linked to inflammatory monocyte and neutrophil recruitment and has been implicated in experimental intestinal inflammation [88,89]. Although both MIP-1α and MIP-1β signal primarily through CCR5, their divergent associations in our models suggest that they may reflect distinct immunological processes, with MIP-1α preferentially marking the myeloid- and Th1/IL-23-driven inflammatory environment characteristic of CD, whereas MIP-1β appears more closely linked to the immune profile observed in UC [90]. Interestingly, the association of MIP-1α with UC varied according to GM-CSF levels and vice versa. Both mediators exert opposing effects on proliferation of hematopoietic stem cells and on myeloid differentiation [91,92,93]. The GM-CSF–MIP-1α interaction in model 3 likely reflects the combined influence of these mediators within a broader inflammatory network rather than independent effects. As such, this statistical interaction should be viewed as an exploratory adjusted association rather than evidence of a direct mechanistic relationship.

Whereas UC appears to be characterized by pathways promoting epithelial maintenance, mucosal healing, and restoration of barrier integrity, CD exhibits stronger signatures of stromal remodeling, angiogenesis, fibrosis, and neuroimmune activation. This pattern was evident in the negative association of SDF-1α, β-NGF, and LIF with the odds of UC, indicating a closer relationship with CD. Among these, SDF-1α is particularly noteworthy because it acts as a potent chemoattractant for fibrocytes and has been reported to be elevated specifically in CD rather than UC [34]. Fibrocytes contribute to intestinal fibrosis through differentiation into fibroblasts, myofibroblasts, and adipocyte-like cells, implicating the SDF-1α/CXCR4 axis in fibrotic remodeling. Moreover, SDF-1α is regulated by the angiogenic transcription factor HIF-1α and promotes recruitment of stem and progenitor cells to injured tissues while providing mitogenic and pro-survival signals to microvascular endothelial cells [94,95]. Thus, the association of SDF-1α with CD is consistent with the greater propensity of this disease toward transmural injury, fibrosis, and vascular remodeling.

The βNGF further supports the involvement of tissue-remodeling pathways in CD. Although best known as a neurotrophic factor, βNGF also exerts important immunomodulatory and profibrotic effects and has previously been linked with increased CD risk by genetic studies [39]. Enteric neuronal alterations are reportedly more extensive in CD than UC [96], and enhanced NGF/tropomyosin receptor kinase A (TrkA) signaling has been demonstrated in Meissner’s plexus, particularly in CD [97]. Moreover, a negative association between βNGF, a survival factor, and UC agrees well with accelerated apoptosis of gangliocytes observed in this IBD phenotype [98]. Beyond neuroimmune interactions, βNGF has been implicated in fibrotic remodeling by promoting collagen and calcium deposition, expression of α-smooth muscle actin, and profibrotic reprogramming of mesenchymal stromal cells [99,100,101]. Its association with CD may therefore reflect both increased neuroimmune activation and enhanced fibrogenesis. This interpretation is further supported by the characteristic expansion of mesenteric adipose tissue (“creeping fat”) in CD, a tissue compartment capable of producing numerous inflammatory, angiogenic, and profibrotic mediators, including IL-10, TNFα, IL-6, IL-1β, IFNγ, IL-8, FGF2, VEGF-A, and RANTES [82,102,103,104,105,106], many of which were associated with CD discrimination in our study.

LIF represents another intriguing component of this CD-associated signature. Although previously reported to exert protective effects in intestinal inflammation [107], LIF is increasingly recognized as a mediator of angiogenesis and fibrotic remodeling in other tissues. It can induce expression of pro-angiogenic VEGF-A, IL-8, and HGF [108,109,110] and has recently been identified as an amplifier of pulmonary fibrosis [111]. Its association with CD may therefore reflect activation of pathways involved in chronic tissue remodeling rather than acute mucosal inflammation. Notably, several additional mediators negatively associated with the odds of UC through their incorporation in PC1, including VEGF-A, FGF2, and IL-8, are key regulators of angiogenesis and fibrosis [112]. Although pathological angiogenesis occurs in both major IBD phenotypes, experimental and genetic evidence suggests a more prominent mechanistic contribution to CD pathogenesis [113]. In this context, the composition of PC1 appears to capture a broader biological program characterized by angiogenesis, stromal activation, and chronic tissue remodeling. The absence of effective anti-fibrotic therapies for CD and the growing interest in interventions targeting broader inflammatory networks rather than individual cytokines [30,114] further underscore the potential biological and therapeutic relevance of these findings.

To our knowledge, this study extends previous work by combining broad cytokine profiling with multivariable modeling to identify immune signatures that differentiate active UC from CD. Beyond established cytokines, we examined an extended panel of less-studied mediators, evaluated interaction effects, and incorporated principal component analysis to account for highly correlated analytes while preserving biological information. This approach reflects the network-like nature of inflammatory signaling better than analyses based solely on individual mediators.

### Study Limitations

Despite encouraging internal discrimination, the models should be viewed as exploratory immune-signature models rather than clinically applicable prediction tools. Their primary aim was to assess whether circulating mediators of inflammation and immunity jointly contain information relevant to distinguishing active UC from active CD. An independent validation cohort was not available in the present study. Moreover, because cross-validation was performed after model structure and feature selection had been established, it provides an internal post-selection assessment of discrimination rather than validation of the complete model-development procedure. Accordingly, the reported AUC, sensitivity, and specificity estimates should not be interpreted as estimates of expected performance in independent patients or as evidence of model transportability or clinical generalizability. This limitation is particularly relevant to the extended-panel model derived from the smaller dataset B. In addition, clinical variables such as disease location, extent, treatment, inflammatory burden, and endoscopic findings were not included and, thus, part of the observed discriminatory signal may reflect differences in clinical case-mix between the UC and CD groups. The findings should therefore be regarded primarily as hypothesis-generating evidence that combinations of circulating immune mediators may capture biologically meaningful differences between active UC and CD. Independent validation in larger, independently recruited cohorts representing broader clinical settings will be required to determine the stability, transportability, and potential clinical utility of these signatures. A particularly informative future validation design would involve prospective application of a prespecified cytokine-based signature in patients with an initially uncertain or IBD-unclassified diagnosis, followed by assessment of classification accuracy once the final clinical diagnosis has been established.

## 4. Materials and Methods

### 4.1. Patients

The study population comprised 138 consecutive patients with active IBD, including 54 individuals diagnosed with UC and 84 with CD, who were hospitalized in the Department of Gastroenterology and Hepatology, Wroclaw Medical University for disease exacerbation. Patients presenting with indeterminate colitis, concomitant severe systemic disorders, malignant diseases, hepatic disorders, or pregnancy were excluded from participation.

Disease activity in CD was assessed using the Crohn’s Disease Activity Index (CDAI), a composite measure integrating clinical symptoms, physical findings, patient-reported outcomes, and relevant medical history. UC activity was evaluated with the Clinical Activity Index (RI; Rachmilewitz Index), which incorporates stool frequency, presence of blood in stools, overall well-being, abdominal pain or cramping, fever, extraintestinal manifestations, and laboratory parameters, including erythrocyte sedimentation rate (ESR) and hemoglobin levels as well as with partial Mayo (pMAYO) encompassing stool frequency, rectal bleeding, and Physician’s Global Assessment subscores. Endoscopic severity of UC was graded according to the Mayo endoscopic subscore (MES). Scores were assigned as follows: 0, inactive disease; 1, mild activity characterized by erythema, reduced vascular pattern, and mild mucosal friability; 2, moderate activity with marked erythema, absent vascular pattern, friability, and erosions; and 3, severe disease marked by spontaneous bleeding and ulceration.

There were 46% males in the CD group and 59% in the UC group (*p* = 0.141). Based on the median values, the UC group was characterized by 23.3% higher age (*p* = 0.007), 2-fold lower CRP concentration (*p* = 0.014), 14.6% higher albumin concentration (*p* = 0.017), 1.5-fold higher total cholesterol concentration (*p* = 0.013), and 28.4% higher high-density lipoprotein (HDL) cholesterol concentration (*p* = 0.040). Regarding the CD group, the lesions were predominantly reported in both ileum and colon (54.3%), and ileum and caecum (11.4%), with ‘colon-only’ cases constituting 20% of the records. All UC patients underwent surgery, as opposed to 66.7% in the CD group (*p* = 0.005). The difference in body mass index (BMI) between these groups was on the verge of statistical significance (*p* = 0.051). The characteristics are shown in Table 6.

The study was conducted using archived samples collected from IBD patients prior to the widespread use of biologics, and none of patients received biologic therapy during the study. Most patients in both UC and CD groups received treatment with 5-aminosalicylate (5-ASA) preparations. In addition, 58% of CD and 62% of UC patients (*p* = 0.727) were administered corticosteroids, 52% of CD and 32% of UC patients (*p* = 0.112) were administered azathioprine with 38% of CD and 29% of UC patients (*p* = 0.526) receiving both. Almost all patients treated with corticosteroids received these drugs in connection with a flare of their condition, which was the reason for their hospitalization. Only 14% of patients with CD and 3% with UC had been treated with azathioprine prior to hospitalization. In the remaining patients, treatment was initiated during hospitalization after sample collection. Univariate analysis of patients treated and not treated with azathioprine showed no significant differences in cytokine concentrations while VEGF-A concentrations were significantly higher (*p* = 0.006) in CD patients treated with corticosteroids.

Smoking status may affect immune pathways in IBD patients [115]. However, except for four patients, none of the participants in the study group smoked cigarettes; therefore, its impact has not been investigated.

### 4.2. Analytical Methods

Peripheral blood samples were collected by venipuncture prior to any treatment and left undisturbed at room temperature for 30 min to allow coagulation. Subsequently, the samples were centrifuged at 720× *g* for 15 min, after which the serum fraction was separated, divided into aliquots, and stored at −80 °C until further analysis.

Concentrations of circulating cytokines, chemokines, and growth factors in serum were determined in duplicate using a bead-based multiplex flow cytometric assay. Measurements were performed on the Bio-Plex 200 system equipped with a high-throughput fluidics module (Bio-Rad, Hercules, CA, USA) and based on Luminex xMAP^®^ technology. Magnetic microspheres coated with specific monoclonal antibodies were employed in commercially available multiplex panels enabling the simultaneous quantification of IL-1β, IL-1RA, IL-2, IL-4, IL-5, IL-6, IL-7, IL-8, IL-9, IL-10, IL-12p70, IL-13, IL-15, IL-17, eotaxin-1, FGF2, G-CSF, GM-CSF, IFNγ, IP10, MCP-1, MIP-1α, MIP-1β, TNFα, VEGF-A, PDGF-BB, and RANTES (27-plex Human Cytokine Group I Assay) and IL-1β, IL-2RA, IL-3, IL-12p40, IL-16, IL-18, CTACK, GRO, HGF, IFNα, LIF, MCP-3, M-CSF, MIF, MIG, β-NGF, CSF, SCGF-β, SDF-1α, TNFβ, and TRAIL (21-plex Human Cytokine Group II Assay), following the manufacturer’s recommendations. The concentrations of IL-1α, IL-2, and TNFβ were below detection limit of the assay and thus these cytokines together with IL12p40 and MCP-3 (many observations below the detection limit) were excluded from analyses. Analyte concentrations were calculated from standard curves generated using four- or five-parameter logistic (4-PL or 5-PL) regression models and processed with Bio-Plex Manager version 6.0 software (Bio-Rad).

### 4.3. Data Analysis

Data preprocessing and analysis were performed using R 4.4.2, including the tidyverse 2.0.0, mgcv 1.9-1, cluster 2.1.8, factoextra 1.0.7, and emmeans 1.10.7 packages, and Statistica 13.3 under the Wroclaw Medical University license. Statistical inference was based on a frequentist framework, with two-sided *p*-values below 0.05 considered statistically significant.

Because many variables, especially circulating immune mediators, were characterized by skewed distributions and/or right-sided tails resulting from extremely high values, the Mann–Whitney U test was used for univariate between-group comparisons of quantitative variables. The chi-square test was used for univariate group-wise comparisons of qualitative variables.

The multivariate association between candidate variables and the odds of active UC, with active CD used as the reference group, was assessed using logistic regression. Because of the skewed distributions and long right-sided tails of many mediator variables, all mediator variables used in multivariable modelling were right-sided Winsorized at the 90th percentile; thus, the most extreme high values were replaced by the corresponding 90th percentile value. This step was applied to reduce the influence of extreme observations on the estimated regression coefficients and should not be interpreted as defining biological thresholds. In addition, continuous variables were median-centered before model fitting to improve model convergence and to make model intercepts and conditional main effects interpretable for a typical patient profile. For variables labelled as analysed per 100 units, the rescaling was performed before model fitting; therefore, the corresponding odds ratios refer directly to a 100-unit increase in the original measurement scale.

Because the extended mediator panel was available only in a subgroup of patients, two related modelling strategies were used. Detailed preprocessing decisions, feature-reduction steps, model-building paths, interaction screening, principal component analysis, internal performance assessment, and sensitivity analyses are provided in the Supplementary Materials, which were prepared as an analytical audit trail for the exploratory modelling workflow.

The first modeling approach used 138 records containing full information on sex, age, IL-1β, IL-1RA, IL-4, IL-5, IL-6, IL-7, IL-8, IL-9, IL-10, IL-12p70, IL-13, IL-15, IL-17, eotaxin-1, FGF2, G-CSF, GM-CSF, IFNγ, IP10, MCP1, MIP1α, MIP1β, TNFα, VEGFα, PDGF-BB, and RANTES. In this approach, feature reduction for the initial multivariable models was performed using stepwise logistic regression. Inclusion and elimination/re-inclusion decisions were based on score and Wald test *p*-values, respectively, with a *p*-value threshold of 0.05. This procedure yielded two preliminary models. A third model was then developed from the second model by including an interaction term between GM-CSF and MIP1α. This interaction was selected after likelihood-ratio screening of candidate two-way interactions between variables retained in the second model.

The assumption of linearity between continuous variables and the log-odds of active UC was assessed during model diagnostics. The Box-Tidwell test suggested a potentially nonlinear association between IL1β and the odds of active UC. To evaluate whether accounting for this nonlinearity improved model fit, a generalized additive logistic model with a spline term for IL1β was fitted. Because the effective degrees of freedom for this term were close to linearity and the spline model did not materially improve residual deviance compared with the simpler linear specification, the model with a linear IL1β term was retained for interpretation. This model also showed better goodness-of-fit than the two preliminary models and was selected as the final Dataset A model.

The second modeling approach used 67 records containing all variables from the first approach, with the addition of IL-2RA, IL-3, IL-12p40, IL-16, IL-18, CTACK, GRO, HGF, IFNα, LIF, MCP3, M-CSF, MIF, MIG, β-NGF, SCF, SCGF-β, SDF1α, and TRAIL. Direct stepwise modelling of the full extended panel was not feasible because of high dimensionality and strong collinearity among several mediators. Therefore, variables with high pairwise correlations, defined as Pearson’s r > 0.80, were first subjected to hierarchical clustering using Ward’s method. The optimal number of clusters was selected based on silhouette width. MCP3 and IL-12p40 were not used for PCA construction because of predominant values near or below the assay detection limit. The remaining correlated mediators were standardized and summarized using principal component analysis. Three principal components were retained based on the elbow criterion and together explained 83.07% of the variance among variables included in the PCA. These principal components, together with sex, age, and mediators not summarized by PCA, were then used as candidates in stepwise logistic regression. This procedure yielded the final exploratory extended-panel model.

Internal discriminative performance of the multivariable models was assessed using 10-fold cross-validation after the model structures had been selected. Analyte concentrations were entered into the multivariable logistic regression models as continuous predictors; therefore, the models did not rely on variable-specific concentration cut-offs, and classification was based on the combined model-derived probability. The evaluated metrics included learning and testing AUC, as well as testing sensitivity and specificity at the threshold maximizing Youden’s J index. Because cross-validation was performed after feature selection, these metrics should be interpreted as internal post-selection estimates of discrimination and not as external validation of the full model-building workflow. No independent validation cohort was available in the present study; therefore, the cross-validation results were used solely to characterize internal discrimination and do not establish model transportability or clinical generalizability. In this context, “learning” and “testing” refer to the training and held-out folds within the cross-validation procedure, respectively, and not to separate development and independent validation cohorts.

The ROC analysis for individual markers was conducted using MedCalc^®^ Statistical Software version 23.5.2 (MedCalc Software Ltd., Ostend, Belgium; https://www.medcalc.org; 2026) licensed to Małgorzata Krzystek-Korpacka. The following interpretational criteria for markers’ overall accuracy based on AUC values were adopted from Çorbacıoğlu et al. [116]: 0.5 ≤ AUC < 0.6 fail; 0.6 ≤ AUC < 0.7 poor; 0.7 ≤ AUC < 0.8 fair; 0.8 ≤ AUC < 0.9 considerable (good); 0.9 ≤ AUC excellent.

## 5. Conclusions

In summary, the cytokine signatures identified in this study support distinct immunopathological profiles underlying active UC and CD. Active UC was characterized predominantly by mediators associated with eosinophil-, mast cell-, and neutrophil-driven mucosal inflammation together with tissue repair and counter-regulatory responses, including eotaxin-1, IL-5, GRO/CXCL1, MIP-1β, SCF, GM-CSF, IL-1RA, TRAIL, SCGFβ, and CTACK. By contrast, active CD was associated with mediators linked to Th1/Th17 immunity, myeloid activation, fibrosis, angiogenesis, and neuroimmune remodeling, including IL-1β, IL-12p70, IL-15, RANTES, MIP-1α, SDF-1α, β-NGF, and LIF.

Beyond confirming established differences between UC and CD, our findings identify several relatively understudied mediators—notably SCGFβ, CTACK, TRAIL, SCF, MIP-1β, SDF-1α, β-NGF, and LIF—as potentially informative components of disease-specific inflammatory networks. Together, these findings broaden our understanding of IBD immunopathology and highlight candidate pathways that warrant further mechanistic investigation and evaluation as potential therapeutic targets. The results further support the concept that information distinguishing IBD phenotypes is more likely to emerge from integrated immune signatures than from individual biomarkers considered in isolation. The proposed signatures remain exploratory and require independent validation. Nevertheless, they provide hypothesis-generating evidence that circulating mediators of inflammation and immunity contain biologically informative patterns that may ultimately contribute to future clinically integrated approaches to IBD classification.

## Figures and Tables

**Table 1:** Concentrations of circulating cytokines, chemokines, and growth factors in IBD patients.

Variable	Active CD			Active UC			*p* ^2^
	Me ^1^	1st Q ^1^	3rd Q ^1^	Me ^1^	1st Q ^1^	3rd Q ^1^	
Eotaxin-1 ^3^	107.98	64.61	157.73	159.66	117.8	209.7	<0.0001
MIP-1β ^3^	89.69	69.14	112.61	111.38	96.61	145.73	0.0001
MCP-1 ^3^	66.09	42.355	104.27	106.81	65.47	156.12	0.001
TRAIL ^4^	66.97	39.75	83.97	79.78	65.55	123.46	0.005
CTACK ^4^	433.33	351.8	540.41	555.46	433.33	641.96	0.010
VEGFA ^3^	120.04	66.33	225.3	194.78	120.15	253.12	0.027
SCF ^4^	108.07	85.83	126.55	132.88	90.66	161.7	0.032
SCGFβ ^4^	12,654	7664	16,857	15,973	11,126	18,912	0.039
M-CSF ^4^	7.37	5.56	14.25	12.56	8.67	19.26	0.056
IFNγ ^3^	85.91	50.04	166.73	96	69.9	304.68	0.057
HGF ^4^	632.57	465.22	816.86	943.76	495.14	1063.71	0.063
GRO ^4^	206.24	145.89	284.83	254.09	183.31	357.35	0.088
IL-13 ^3^	15.82	10.49	23.08	19.09	11.32	28.27	0.097
IL-4 ^3^	4.09	2.845	7.41	5.7	2.98	10.15	0.117
IP10 ^3^	735.9	535.5	964.1	865.5	584.6	1194.3	0.122
IL-1RA ^3^	188.1	120.8	274.2	211.4	114.8	428.1	0.135
IL-17 ^3^	153.2	90.4	232.9	181.5	112.6	246.3	0.165
IL-5 ^3^	8.49	4.85	12.32	9.84	5.84	14.62	0.224
IL-18 ^4^	90.3	56.44	108.1	96.9	67.2	172.7	0.232
IL-15 ^3^	23.13	14.97	39.44	29.88	17.28	48.72	0.270
IL-8 ^3^	55.16	39.8	91.32	61.9	42.43	117.8	0.301
GM-CSF ^3^	39.17	28.245	51.92	44.21	26.36	74.08	0.318
PDGF-BB ^3^	3639	2601	54,277	3806	2675	6688	0.318
IL-9 ^3^	26.75	19.38	43.17	30.71	20.5	45.93	0.344
TNFα ^3^	36.16	23.32	55.1	42.14	21.38	68.25	0.364
IL-12p40 ^4^	154.33	50.2	286.52	192.51	80.17	346.75	0.446
IL-10 ^3^	18.98	11.8	31.39	20.91	13.13	34.9	0.448
FGF2 ^3^	72.06	53.585	110.56	74.37	58.44	111.96	0.448
IL-2RA ^4^	149.59	115.15	207.94	167.55	120.69	194.27	0.541
IL-3 ^4^	87.99	65.65	127.26	121.66	63.73	180.23	0.575
LIF ^4^	12.4	7.9	18.6	14.87	7.66	26.16	0.585
IL-6 ^3^	22.75	15.15	40.15	21.76	13.31	37.46	0.646
IL-12p70 ^3^	65.78	42.595	128.43	77.07	40.6	119.06	0.662
IL-16 ^4^	262.05	196.76	359.42	266.31	224.15	376.17	0.723
MIF ^4^	811.54	490.3	1477.03	835.89	467.34	1525.24	0.723
MIP-1α ^3^	4.37	3.06	6.59	5.04	3.26	6.45	0.743
βNGF ^4^	3	2.13	3.99	2.87	1.86	4.01	0.752
SDF-1α ^4^	100.3	84.69	111.92	104.84	85.34	125.19	0.772
IFNα ^4^	30.31	26.92	37.35	31.64	26.02	38.49	0.872
RANTES ^3^	10,786	4510	15,926	9959	6890	14,375	0.874
G-CSF ^3^	80.34	49.875	125.91	73.85	49.71	138.65	0.915
MIG ^4^	1253	948	1793	1307	723	2194	0.933
IL-7 ^3^	15.82	12.495	21.57	16.68	11.48	26.71	0.984
IL-1β ^3^	2.31	1.51	4.73	2.43	1.23	5.59	1
MCP-3 ^4^	15.59	1.64	27.04	15.16	6.6	22.24	1

^1^, expressed in pg/mL; ^2^, data analyzed using Mann–Whitney U test; ^3^, determined in patients from dataset A (84 CD and 54 UC); ^4^, determined in patients from subset B (42 CD and 25 UC); CD, Crohn’s disease; UC, ulcerative colitis; Me, median value; Q, quartile; MIP, macrophage inflammatory protein; MCP, monocyte chemoattractant protein; TRAIL, tumor necrosis factor-related apoptosis-inducing ligand; CTACK, cutaneous T-cell-attracting chemokine; VEGFA, vascular endothelial growth factor A; SCF, stem cell factor; SCGFβ, stem cell growth factor-β; M-CSF, macrophage colony-stimulating factor; IFN, interferon; HGF, hepatocyte growth factor; GRO, growth-regulated oncogene; IL, interleukin; IP10, interferon gamma-induced protein 10 kDa; IL-1RA, interleukin-1 receptor antagonist; GM-CSF, granulocyte-macrophage colony-stimulating factor; PDGF-BB, platelet-derived growth factor-BB; TNFα, tumor necrosis factor α; FGF2, fibroblast growth factor 2; IL-2RA, interleukin-2 receptor antagonist; LIF, leukemia inhibitory factor; MIF, macrophage migration inhibitory factor; βNGF, nerve growth factor β; SDF-1α, stromal cell-derived factor 1α; RANTES, ‘regulated upon activation, normal T-cell expressed, and secreted’; G-CSF, granulocyte colony-stimulating factor; MIG, monokine induced by gamma interferon.

**Table 2:** Discriminatory power of selected individual chemokines, cytokines, and growth factors in differentiating between active CD and UC.

Variable	AUC (95% CI)	*p*	Cut-Off ^1^	Sens.	Spec.	LR+	LR−	J
CRP	0.68 (0.55–0.79)	0.009	≤25.7	71%	64.7%	2.01	0.45	0.357
Eotaxin-1	0.71 (0.62–0.78)	<0.001	>138.23	70.4%	67.1%	2.14	0.44	0.374
MIP-1β	0.70 (0.62–0.77)	<0.001	>89.11	85.2%	50%	1.70	0.30	0.352
MCP-1	0.66 (0.58–0.74)	<0.001	>105.24	51.9%	79.8%	2.56	0.60	0.316
TRAIL	0.70 (0.58–0.81)	0.002	>87.67	48%	83.3%	2.88	0.62	0.313
CTACK	0.69 (0.56–0.80)	0.005	>548.72	52%	81%	2.73	0.59	0.330
VEGF-A	0.61 (0.53–0.69)	0.021	>116.15	75.9%	50%	1.52	0.48	0.259
SCF	0.66 (0.53–0.77)	0.038	>130.01	56%	81%	2.94	0.51	0.370
SCGFβ	0.65 (0.53–0.76)	0.026	>9287.5	88%	38.1%	1.42	0.32	0.261

^1^, expressed in pg/mL, except for CRP expressed in mg/L; AUC, area under ROC (receiver operating characteristics) curve; CI, confidence interval; *p*, probability; sens., sensitivity; spec., specificity; LR+/−, positive and negative likelihood ratios; J, Youden index; CRP, C-reactive protein; MIP-1β, macrophage inflammatory protein 1β; MCP-1, monocyte chemoattractant protein 1; TRAIL, tumor necrosis factor-related apoptosis-inducing ligand; CTACK, cutaneous T-cell-attracting chemokine; VEGF-A, vascular endothelial growth factor A; SCF, stem cell factor; SCGFβ, stem cell growth factor-β.

**Table 3:** Multivariable logistic regression model exploring the odds of active UC among active IBD based on dataset A—model 3.

Model 3					
Learning AUC: 0.92 ± 0.022; testing AUC 0.84 ± 0.033; max testing sensitivity: 74%; max testing specificity: 80% (both metrics at Youden’s J = 0.538)					
**Effect/interaction**	**Interpretation**	**Estimate**	**Estimate −95% CI**	**Estimate 95% CI**	***p***
Intercept	Baseline odds of UC for a typical person in the database (all the below chemokines at their median value)	0.4993	0.2478	1.0061	0.052
il1b_winsorized_c	Fold increase in OR upon each one-unit increase in IL-1β	0.1908	0.0797	0.4566	<0.001
il1ra_winsorized_c	Fold increase in OR upon each one-unit increase in IL-1RA	1.0134	1.0051	1.0217	0.002
il4_winsorized_c	Fold increase in OR upon each one-unit increase in IL-4	0.5749	0.3611	0.9154	0.020
il5_winsorized_c	Fold increase in OR upon each one-unit increase in IL-5	1.4782	1.1314	1.9315	0.004
il10_winsorized_c	Fold increase in OR upon each one-unit increase in IL-10	0.9328	0.8864	0.9817	0.008
eotaxin_winsorized_c	Fold increase in OR upon each one-unit increase in eotaxin-1	1.0209	1.0102	1.0317	<0.001
gm_csf_winsorized_c	Fold increase in OR upon each one-unit increase in GM-CSF at the median value of MIP1α	1.0659	1.0298	1.1033	<0.001
ifn_g_winsorized_c	Fold increase in OR upon each one-unit increase in IFNγ	1.0358	1.0167	1.0552	<0.001
mip1a_winsorized_c	Fold increase in OR upon each one-unit increase in MIP1α at the median value of GM-CSF	0.4300	0.2540	0.7280	0.002
mip1b_winsorized_c	Fold increase in OR upon each one-unit increase in MIP1β	1.0535	1.0278	1.0799	<0.001
rantes_winsorized_c	Fold increase in OR upon each 100-unit increase in RANTES	0.9814	0.9690	0.9939	0.004
gm_csf_winsorized_c*mip1a_winsorized_c	Fold change in OR associated with GM-CSF or MIP1α upon each one-unit increase in: MIP1α (in case of OR for GM-CSF) or GM-CSF (in case of OR for MIP1α)	0.9849	0.9730	0.9969	0.014

The ‘_winsorized_c’ term in the names of effects indicate Winsorization of these variables at 90th percentile and subsequently centering them at their median value. CI, confidence interval; *p*, probability; OR, odds ratio; IL, interleukin; GM-CSF, granulocyte-macrophage colony-stimulating factor; MIP, macrophage inflammatory protein; IFNγ, interferon γ; RANTES, ‘regulated upon activation, normal T-cell expressed, and secreted’.

**Table 4:** Multivariable logistic regression model exploring the odds of active UC among active IBD based on dataset B—model 4.

Model 4					
Learning AUC: 0.91 ± 0.037; testing AUC: 0.83 ± 0.056; max testing sensitivity: 64%; max testing specificity: 95.2% (both metrics at Youden’s J = 0.592)					
**Effect**	**Interpretation**	**Estimate**	**Estimate −95% CI**	**Estimate 95% CI**	***p***
Intercept	Baseline odds of UC for a person characterized by centered values of the non-PCA mediators and the reference value of the PCA-derived component.	0.1300	0.0350	0.4826	0.002
il1ra_winsorized_c	Fold increase in OR upon each one-unit increase in IL-1RA	1.0159	1.0045	1.0274	0.006
trail_winsorized_c	Fold increase in OR upon each one-unit increase in TRAIL	1.0755	1.0255	1.1278	0.003
PC1	Fold increase in OR upon each one-unit increase in PC1	9.0876	2.2875	36.1031	0.002
gro_winsorized_c	Fold increase in OR upon each one-unit increase in GRO	1.0314	1.0117	1.0515	0.002
gm_csf_winsorized_c	Fold increase in OR upon each one-unit increase in GM-CSF	1.0988	1.0329	1.1689	0.003
scf_winsorized_c	Fold increase in OR upon each one-unit increase in SCF	1.0340	1.0007	1.0685	0.045
mip1b_winsorized_c	Fold increase in OR upon each one-unit increase in MIP-1β	1.0512	1.0119	1.0919	0.010

The ‘_winsorized_c’ term in the names of effects indicates Winsorization of these variables at the 90th percentile and subsequent centering at their median value. PCA, principal component analysis; CI, confidence interval; *p*, probability; OR, odds ratio; PC1, principal component 1; IL-1RA, IL-1 receptor antagonist; TRAIL, tumor necrosis factor-related apoptosis-inducing ligand; GRO, growth-regulated oncogene; GM-CSF, granulocyte-macrophage colony-stimulating factor; SCF, stem cell factor; MIP, macrophage inflammatory protein.

**Table 5:** Approximate back-projected contribution of the chemokines, cytokines, and growth factors contributing to Principal Component 1 (PC1) and the odds of UC based on revised model 4.

Variable ^1^	Interpretation	EV	β of PC1	βEV	Estimate
mip1a_winsorized_sdized	Approx. model-implied OR contribution per one SD ↑ in MIP-1α along PC1 EV direction	−0.2980	2.2069	−0.6576	0.5181
ifn_a_winsorized_sdized	Approx. model-implied OR contribution per one SD ↑ in IFNα along the PC1 EV direction	−0.2968	2.2069	−0.6550	0.5195
sdf_1a_winsorized_sdized	Approx. model-implied OR contribution per one SD ↑ in SDF-1α along the PC1 EV direction	−0.2956	2.2069	−0.6524	0.5208
b_ngf_winsorized_sdized	Approx. model-implied OR contribution per one SD ↑ in βNGF along the PC1 EV direction	−0.2925	2.2069	−0.6456	0.5243
il3_winsorized_sdized	Approx. model-implied OR contribution per one SD ↑ in IL-3 along the PC1 EV direction	−0.2867	2.2069	−0.6327	0.5312
lif_winsorized_sdized	Approx. model-implied OR contribution per one SD ↑ in LIF along the PC1 EV direction	−0.2830	2.2069	−0.6246	0.5355
vegfa_winsorized_sdized	Approx. model-implied OR contribution per one SD ↑ in VEGF-A along the PC1 EV direction	−0.2491	2.2069	−0.5497	0.5771
il5_winsorized_sdized	Approx. model-implied OR contribution per one SD ↑ in IL-5 along the PC1 EV direction	−0.2481	2.2069	−0.5476	0.5783
fgf2_winsorized_sdized	Approx. model-implied OR contribution per one SD ↑ in FGF2 along the PC1 EV direction	−0.2435	2.2069	−0.5373	0.5843
il15_winsorized_sdized	Approx. model-implied OR contribution per one SD ↑ in IL-15 along the PC1 EV direction	−0.2419	2.2069	−0.5338	0.5864
il8_winsorized_sdized	Approx. model-implied OR contribution per one SD ↑ in IL-8 along the PC1 EV direction	−0.2363	2.2069	−0.5216	0.5936
il12p70_winsorized_sdized	Approx. model-implied OR contribution per one SD ↑ in IL-12p70 along the PC1 EV direction	−0.2266	2.2069	−0.5001	0.6065
g_csf_winsorized_sdized	Approx. model-implied OR contribution per one SD ↑ in G-CSF along the PC1 EV direction	−0.2201	2.2069	−0.4858	0.6152
il4_winsorized_sdized	Approx. model-implied OR contribution per one SD ↑ in IL-4 along the PC1 EV direction	−0.1940	2.2069	−0.4281	0.6517
ifn_g_winsorized_sdized	Approx. model-implied OR contribution per one SD ↑ in IFNγ along the PC1 EV direction	−0.1867	2.2069	−0.4121	0.6622
il1b_winsorized_sdized	Approx. model-implied OR contribution per one SD ↑ in IL-1β along the PC1 EV direction	−0.1354	2.2069	−0.2989	0.7417

^1^, Variables are ranked based on the approximate estimate. The ‘_winsorized_c’ term in the names of effects indicates Winsorization of these variables at the 90th percentile and subsequent centering at their median value, whereas the ‘_sdized’ term indicates standardization. CI, confidence interval; EV, eigenvector, i.e., the raw coefficient used to construct the principal component from standardized variables; approx.; approximate; OR, odds ratio; ↑, increase; SD, standard deviation; MIP, macrophage inflammatory protein; IFN, interferon; SDF, stromal cell-derived factor; βNGF, nerve growth factor β; IL, interleukin; LIF, leukemia inhibitory factor; VEGF, vascular endothelial growth factor; FGF, fibroblast growth factor; G-CSF, granulocyte colony-stimulating factor. The approximated estimates were calculated as exp(βPC1 × EV). They represent approximate back-projected contributions to the PC1-associated direction of the log-odds of active UC and should not be interpreted as independent single-marker regression effects.

**Table 6:** Characteristics of study population (dataset A).

Variable	CD Active (*n* = 84)			UC Active (*n* = 54)			*p*
	Me	1st Q	3rd Q	Me	1st Q	3rd Q	
Age	31	27	41	37	30	50	0.007
CDAI	239	190	310	-	-	-	-
pMAYO	-	-	-	7	5	9	-
RI	-	-	-	10	7	15	-
ESR	30	18	52	25	10	60	0.393
CRP	31.7	16.9	94.5	15.6	2.5	65.4	0.014
ALP	72	60	95	69	62	96	0.847
ASPAT	14	11	19	17	13	23	0.067
ALAT	13	8	19	15	11	24	0.085
GGTP	18.6	16	25.6	26.4	21	40	0.021
TP	7.3	6.34	7.7	6.9	6.5	7.3	0.239
ALB	3.64	3.05	4.16	4.17	3.90	4.38	0.017
BMI	21.47	19.38	23.51	22.65	21.77	25.15	0.051
HGB	12.3	10.8	13.3	12.45	11.0	14.1	0.456
HCT	38.1	34.7	41	38.15	33.4	43.3	0.663
RBC	4.55	4.10	4.94	4.48	3.77	4.9	0.807
WBC	8.15	6.21	11.30	7.73	6.15	9.02	0.097
PLT	366	316	551	318	283	434	0.355
MCV	85	76.2	89.6	87	82.2	92	0.121
MCHC	32.3	30.4	32.9	32.6	31.9	33.4	0.083
Tc	130	114	192	195	138	231	0.013
HDLc	48.3	43.7	63.1	62	49.1	71.7	0.040
LDLc	78.95	63.7	141	108	79	138.5	0.377
TG	89	55	114	105	84	110	0.299

CD, Crohn’s disease; UC, ulcerative colitis; Me, median value; Q, quartile; *n*, number of observations; CDAI, Crohn’s disease activity index; pMAYO; partial Mayo score (without endoscopy); RI, Rachmilewitz index; ESR, erythrocyte sedimentation rate; CRP, C-reactive protein in mg/L; ALP, alkaline phosphatase in IU/L; ASPAT, aspartate aminotransferase in IU/L; ALAT, alanine aminotransferase in IU/L; GGTP, γ-glutamyl transpeptidase in IU/L; TP, total protein in g/L; ALB, albumin in g/L; BMI, body mass index in kg/m^2^; HGB, hemoglobin in g/dL; HCT, hematocrit in %; RBC, red blood cell count [×10^6^/μL]; WBC, white blood cell count [×10^3^/μL]; PLT, platelet count [×10^3^/μL]; MCV, mean corpuscular volume in fL; MCHC, mean corpuscular hemoglobin concentration in g/dL; tCHOL, total cholesterol in mg/dL; HDLc, cholesterol of high density lipoprotein fraction in mg/dL; LDLc, cholesterol of light density lipoprotein fraction in mg/dL; TG, triglicerides in mg/dL.

## Data Availability

Dataset available on request from the authors.

## Supplementary Materials

The following supporting information can be downloaded at https://www.mdpi.com/article/10.3390/ijms27167217/s1: details on the exploratory multivariable modeling, including preprocessing decisions, feature reduction procedures, model-building steps, internal performance assessment, and sensitivity analyses.

## Abbreviations

The following abbreviations are used in this manuscript:

5-ASA	5-aminosalicylate
AUC	Area under ROC curve
BMI	Body mass index
CCR	C-C motif chemokine receptor
CD	Crohn’s disease
CI	Confidence interval
CRP	C-reactive protein
CTACK	Cutaneous T-cell-attracting chemokine (CCL27)
ESR	Erythrocyte sedimentation rate
EV	Eigenvector
FGF2	Fibroblast growth factor 2
G-CSF	Granulocyte colony-stimulating factor
GM-CSF	Granulocyte-macrophage colony-stimulating factor
GRO	Growth-regulated oncogene (CXCL1)
HDL	High-density lipoprotein
HGF	Hepatocyte growth factor
HLA	Major histocompatibility complex
IBD	Inflammatory bowel disease
IBDU	Inflammatory bowel disease unclassified
IFN	Interferon
IL	Interleukin
IL-1ra	Interleukin-1 receptor antagonist
ILCs	Innate lymphoid cells
IP-10	Interferon gamma-induced protein 10 kDa
J	Youden index
LIF	Leukemia inhibitory factor
LR	Likelihood ratio
MCP	Monocyte chemoattractant protein
M-CSF	Macrophage colony-stimulating factor
MES	Mayo endoscopic subscore
MIF	Macrophage migration inhibitory factor
MIG	Monokine induced by gamma interferon
MIP	Macrophage inflammatory protein
β-NGF	Nerve growth factor β
OR	Odds ratio
PC	Principal component
PCA	Principal component analysis
PDGF-BB	Platelet-derived growth factor-BB
pMAYO	Partial Mayo score
RANTES	‘Regulated upon activation, normal T-cell expressed, and secreted’ (CCL5)
RI	Rachmilewitz Index
ROC	Receiver operating characteristics
SCF	Stem cell factor
SCGFβ	Stem cell growth factor-β (CLEC11A)
SD	Standard deviation
SDF-1α	Stromal cell-derived factor 1α (CXCL12)
Sens.	Sensitivity
Spec.	Specificity
TNF-α	Tumor necrosis factor α
TRAIL	Tumor necrosis factor-related apoptosis-inducing ligand
TrkA	Tropomyosin receptor kinase A
UC	Ulcerative colitis
VEGF-A	Vascular endothelial growth factor A
