# Supplementary material for: Inflammatory and Immune Cytoprofiles of Active Ulcerative Colitis from Crohn’s Disease—Insights from Multivariable Modeling
Source: Int J Mol Sci. 2026 Aug 13;27(16):7217. doi: 10.3390/ijms27167217 (PMC13513676; doi:10.3390/ijms27167217)
Supplement: Supplementary file 1 [file ijms-27-07217-s001.zip › ijms-4482422-supplementary.pdf]

Supplementary Materials

Purpose and scope of the Supplementary Materials

These Supplementary Materials provide an analytical audit trail for the exploratory multivariable modelling of circulating immunomodulators used to discriminate between active ulcerative colitis (UC) and active Crohn's disease (CD). The document summarizes preprocessing decisions, feature-reduction procedures, model-building steps, internal performance assessment, and sensitivity analyses.

The analyses were designed as exploratory discriminative models. They were not intended to establish causal effects of individual mediators, nor to provide an externally validated diagnostic classifier. The primary aim was to assess whether multivariable circulating immunomodulator profiles contained a signal distinguishing active UC from active CD in the available cohort.

No model was re-estimated during preparation of these Supplementary Materials. The tables reorganize and standardize the outputs generated during the original analysis.

For completeness and traceability, the final specifications of Models 3 and 4 (final models shown in the main body of the manuscript) are reproduced here with full coefficient-level details and interpretational notes.

1 Overview of analytical datasets

Two related analytical datasets were used. Dataset A included the full active IBD cohort with the common mediator panel. Dataset B included a smaller subgroup with an extended mediator panel. Therefore, results from Dataset B should be interpreted as an extended-panel exploratory analysis rather than as an independent validation of Dataset A.

Table S1: Overview of analytical datasets and their role in model development.

Dataset	N	Clinical comparison	Mediator panel	Analytical role
Dataset A	138	Active UC vs. active CD	Common circulating cytokine, chemokine, and growth factor panel	Main exploratory discriminative modelling dataset; basis for Models 1–3.
Dataset B	67	Active UC vs. active CD	Extended mediator panel available in a subgroup	Extended-panel exploratory analysis; basis for Model 4; particularly sensitive to dimensionality and collinearity.

2 Preprocessing and interpretation of model terms

Continuous mediator concentrations used in logistic regression models were right-sided winsorized at the 90th percentile to reduce the influence of extreme high values. This preprocessing step was pragmatic and should not be interpreted as defining biological thresholds. After winsorization, variables were median-centered before model fitting; therefore, model intercepts and conditional main effects refer to a profile with continuous predictors at their sample medians.

For variables labelled “per 100 units”, the variable was rescaled before model fitting. The reported odds ratio and confidence interval therefore correspond directly to a 100-unit increase in the original measurement scale.

In Dataset B, highly correlated mediators were summarized using principal component analysis (PCA). Variables entering PCA were standardized before component extraction. Principal components should be interpreted as aggregate mediator profiles rather than as individual biomarkers.

3 General analytical decisions

The original data required a pragmatic modelling workflow because the mediator distributions were skewed, some markers had extreme high values, the number of candidate predictors was large relative to the sample size, and several mediators were strongly correlated. The following table summarizes the main analytical problems, applied decisions, and their interpretational consequences.

Table S2: Summary of analytical problems, decisions, and interpretational consequences.

Problem encountered	Decision applied	Dataset affected	Interpretational consequence
Skewed mediator distributions with high right tails	Right-sided winsorization at the 90th percentile	Dataset A, Dataset B	Coefficients describe associations after limiting the influence of extreme high observations. This improves robustness but does not define a biological cut-off.
Non-interpretable zero values for continuous mediator concentrations	Median centering after winsorization	Dataset A, Dataset B	Model intercepts and main effects are interpreted around a typical patient profile, with continuous variables equal to their sample medians.
High dimensionality relative to sample size	Exploratory feature-reduction procedure based on stepwise model-building	Dataset A, Dataset B	Selected terms should be interpreted as candidate discriminative signals, not as confirmed independent biomarkers.
Potentially relevant two-way interactions	Likelihood-ratio screening of candidate interactions after Model 2	Dataset A	The GM-CSF $\times$ MIP-1α interaction was retained in Model 3 based on the interaction-screening procedure.
High collinearity between mediators in the extended panel	Hierarchical clustering followed by principal component analysis for highly correlated variables	Mainly Dataset B	Correlated mediators were summarized by latent components to reduce collinearity and improve model feasibility. Principal components should be interpreted as aggregate profiles, not as individual biomarkers.
Markers with problematic values near or below the limit of detection	Exclusion from PCA construction and the final PCA-based modelling workflow where appropriate	Dataset B	Reduced technical instability, but narrowed the set of extended-panel mediators represented in the PCA-based extended-panel model.
Concern about possible nonlinearity of a selected mediator effect	Spline sensitivity analysis for IL-1β in Model 3	Dataset A	Used to assess whether a nonlinear smooth term materially improved fit compared with the linear specification.

Table S2: Summary of analytical problems, decisions, and interpretational consequences continued.

Problem encountered	Decision applied	Dataset affected	Interpretational consequence
Model performance assessment after feature selection	Feature selection was performed once, followed by 10-fold cross-validation of the selected model structure	Dataset A, Dataset B	Cross-validation metrics should be interpreted as post-selection internal performance estimates. They do not estimate the performance of the full feature-selection procedure and do not replace external validation.

Interpretational note. Because model-building involved feature reduction performed before cross-validation, the reported p-values and cross-validation metrics should be interpreted cautiously. The models summarize candidate immune mediator profiles associated with discrimination between active UC and active CD in this cohort. They should not be interpreted as causal models or externally validated diagnostic tools.

4 Dataset A: common mediator panel

4.1 Candidate variables and preprocessing

Dataset A contained 138 records with complete information for the common mediator panel and was used as the main exploratory modelling dataset. The initial set of candidate variables included sex, age, and the circulating immunomodulators listed in Table S3. Mediator variables used in modelling were winsorized at the 90th percentile and then centered at their median value.

Table S3: Candidate variables considered in Dataset A.

Variable group	Variables
Clinical/demographic variables	Sex, age
Cytokines and interleukins	IL-1β, IL-1RA, IL-4, IL-5, IL-6, IL-7, IL-8, IL-9, IL-10, IL-12p70, IL-13, IL-15, IL-17, IFNγ, TNFα
Chemokines	Eotaxin, IP10, MCP-1, MIP-1α, MIP-1β, RANTES
Growth factors and related mediators	FGF2, G-CSF, GM-CSF, VEGF-A, PDGF-BB

4.2 Model 1: stepwise inclusion summary

Model 1 was developed using stepwise inclusion with a p-value cut-off of 0.05. Table S4 summarizes only the model-changing inclusion decisions. The full stepwise trace contained candidate variables that were tested but not retained; those rows are not reproduced in the main flow of the Supplementary Materials to preserve readability.

4.3 Model 2: elimination and re-inclusion summary

Model 2 was developed using an elimination/re-inclusion strategy. Table S5 summarizes terms removed during backward elimination. The final retained variables are shown together with all models in Table S9.

Table S4: Condensed stepwise inclusion path for Model 1 in Dataset A.

Step	Term added	Selection statistic	p-value	Comment
1	Eotaxin	Score $\chi^2 = 17.391$	< 0.0001	First mediator retained in the model.
2	MIP-1β	Score $\chi^2 = 12.336$	0.0004	Added after eotaxin.
3	MIP-1α	Score $\chi^2 = 5.272$	0.0217	Added after eotaxin and MIP-1β.
4	IFNγ	Score $\chi^2 = 10.216$	0.0014	Added as the fourth mediator.
5	GM-CSF	Score $\chi^2 = 6.248$	0.0124	Added as the fifth mediator.
6	RANTES	Score $\chi^2 = 6.219$	0.0126	Added as the final retained term in Model 1.

Table S5: Condensed elimination path for Model 2 in Dataset A.

Step	Term removed	Wald statistic	Wald p-value	Comment
1	IL-6	0.002	0.9676	Weak contribution.
2	Age	0.007	0.9352	Weak contribution.
3	TNFα	0.056	0.8133	Weak contribution.
4	FGF2	0.052	0.8201	Weak contribution.
5	PDGF-BB	0.153	0.6955	Weak contribution.
6	IL-13	0.228	0.6328	Weak contribution.
7	MCP-1	0.197	0.6573	Weak contribution.
8	IL-7	0.312	0.5767	Weak contribution.
9	IP10	0.701	0.4025	Weak contribution.
10	IL-15	0.800	0.3712	Weak contribution.
11	Sex	0.891	0.3451	Weak contribution.
12	IL-17	0.598	0.4394	Weak contribution.
13	IL-9	2.184	0.1394	Weak contribution.
14	IL-12p70	3.294	0.0695	Borderline during elimination.
15	VEGF-A	2.418	0.1200	Weak contribution.
16	IL-8	3.814	0.0508	Borderline during elimination.
17	G-CSF	3.217	0.0729	Borderline during elimination.

4.4 Interaction screening after Model 2

Candidate two-way interactions between variables retained in Model 2 were screened using likelihood-ratio tests. Table S6 shows interactions with p-values below 0.10. The strongest interaction signal was observed for GM-CSF $\times$ MIP-1α, which was subsequently retained in Model 3.

Table S6: Interaction screening after Model 2 in Dataset A: interactions with $p < 0.10$.

Variable 1	Variable 2	LR χ^2	p-value
GM-CSF	MIP-1α	12.1485	0.0005
IL-10	MIP-1α	5.8805	0.0153

Table S6: Interaction screening after Model 2 in Dataset A continued.

Variable 1	Variable 2	LR χ^2	p-value
MIP-1β	RANTES per 100 units	4.8921	0.0270
GM-CSF	MIP-1β	4.6515	0.0310
GM-CSF	IL-5	4.4799	0.0343
IL-10	MIP-1β	4.4118	0.0357
GM-CSF	IL-4	3.8755	0.0490
IL-1RA	IL-5	3.8557	0.0496
IL-1RA	MIP-1β	3.0442	0.0810
Eotaxin	IL-4	2.9595	0.0854
IL-1β	IL-1RA	2.8754	0.0899

Note. Only the GM-CSF × MIP-1α interaction was retained in Model 3. The remaining screened interactions were not retained in the final main model.

4.5 Comparison of Models 1–3 and spline sensitivity

Model 3 was developed by extending Model 2 with the GM-CSF × MIP-1α interaction. A spline sensitivity analysis was additionally performed for IL-1β, because a possible nonlinearity of its association with the log-odds of UC was suggested during diagnostic checking. The spline variant did not materially improve model fit compared with the simpler linear specification; therefore, Model 3 with a linear IL-1β term was retained for interpretation.

Table S7: Goodness-of-fit comparison of Dataset A models.

Model	Residual df	Residual deviance	df	Deviance difference	p-value
Model 3	125	94.90	–	–	Reference model
Model 2	126	101.87	-1	-6.9724	0.0083
Model 1	131	124.70	-5	-22.8241	0.0004
Model 3 with spline term for IL-1β (EDF = 1.515)	124.48	93.454	0.515	1.4459	0.1082

Table S8: Internal post-selection discrimination metrics for Dataset A models.

Model	Learning AUC	Testing AUC	Sensitivity	Specificity
Model 1	0.859 ± 0.031	0.824 ± 0.035	0.852	0.655
Model 2	0.904 ± 0.024	0.856 ± 0.031	0.907	0.667
Model 3	0.924 ± 0.022	0.841 ± 0.033	0.741	0.798

AUC metrics were obtained after the model structure had been selected. Therefore, they describe post-selection internal discrimination and should not be interpreted as external validation of the full model-building procedure. Model 3 was the final Dataset A model and included the GM-CSF × MIP-1α interaction.

4.6 Final terms of Dataset A models

Table S9 summarizes the final coefficient estimates of Models 1–3. Odds ratios above 1 indicate higher odds of active UC relative to active CD; odds ratios below 1 indicate lower odds of active UC relative to active CD.

Table S9: Final terms of exploratory logistic regression Models 1–3 in Dataset A.

Model	Effect	OR	95% CI	p-value	Interpretation
Model 1	Intercept	0.2848	0.1583–0.5124	< 0.0001	Baseline odds for median-centered mediator profile.
Model 1	Eotaxin	1.0160	1.0074–1.0247	0.0002	Higher eotaxin associated with higher odds of active UC.
Model 1	GM-CSF	1.0323	1.0103–1.0549	0.0038	Higher GM-CSF associated with higher odds of active UC.
Model 1	IFNγ	1.0121	1.0058–1.0185	0.0002	Higher IFNγ associated with higher odds of active UC.
Model 1	MIP-1α	0.4348	0.2944–0.6421	< 0.0001	Higher MIP-1α associated with lower odds of active UC.
Model 1	MIP-1β	1.0409	1.0238–1.0584	< 0.0001	Higher MIP-1β associated with higher odds of active UC.
Model 1	RANTES per 100 units	0.9899	0.9818–0.9980	0.0151	Higher RANTES associated with lower odds of active UC.
Model 2	Intercept	0.4017	0.2050–0.7872	0.0079	Baseline odds for median-centered mediator profile.
Model 2	IL-1β	0.2198	0.0982–0.4921	0.0002	Higher IL-1β associated with lower odds of active UC.
Model 2	IL-1RA	1.0111	1.0037–1.0185	0.0033	Higher IL-1RA associated with higher odds of active UC.
Model 2	IL-4	0.5696	0.3562–0.9109	0.0188	Higher IL-4 associated with lower odds of active UC.
Model 2	IL-5	1.4997	1.1458–1.9630	0.0032	Higher IL-5 associated with higher odds of active UC.
Model 2	IL-10	0.9280	0.8847–0.9734	0.0022	Higher IL-10 associated with lower odds of active UC.
Model 2	Eotaxin	1.0193	1.0091–1.0296	0.0002	Higher eotaxin associated with higher odds of active UC.
Model 2	GM-CSF	1.0564	1.0244–1.0893	0.0005	Higher GM-CSF associated with higher odds of active UC.
Model 2	IFNγ	1.0380	1.0188–1.0575	< 0.0001	Higher IFNγ associated with higher odds of active UC.
Model 2	MIP-1α	0.3194	0.1893–0.5389	< 0.0001	Higher MIP-1α associated with lower odds of active UC.
Model 2	MIP-1β	1.0585	1.0323–1.0853	< 0.0001	Higher MIP-1β associated with higher odds of active UC.
Model 2	RANTES per 100 units	0.9802	0.9684–0.9922	0.0012	Higher RANTES associated with lower odds of active UC.
Model 3	Intercept	0.4993	0.2478–1.0061	0.0520	Baseline odds for median-centered mediator profile.
Model 3	IL-1β	0.1908	0.0797–0.4566	0.0002	Higher IL-1β associated with lower odds of active UC.
Model 3	MIP-1α	0.4300	0.2540–0.7280	0.0017	Conditional main effect at median GM-CSF.
Model 3	IL-4	0.5749	0.3611–0.9154	0.0197	Higher IL-4 associated with lower odds of active UC.
Model 3	IL-10	0.9328	0.8864–0.9817	0.0076	Higher IL-10 associated with lower odds of active UC.
Model 3	RANTES per 100 units	0.9814	0.9690–0.9939	0.0038	Higher RANTES associated with lower odds of active UC.
Model 3	GM-CSF $\times$ MIP-1α	0.9849	0.9730–0.9969	0.0136	Interaction term retained after LR screening.
Model 3	IL-1RA	1.0134	1.0051–1.0217	0.0015	Higher IL-1RA associated with higher odds of active UC.
Model 3	Eotaxin	1.0209	1.0102–1.0317	0.0001	Higher eotaxin associated with higher odds of active UC.
Model 3	IFNγ	1.0358	1.0167–1.0552	0.0002	Higher IFNγ associated with higher odds of active UC.
Model 3	MIP-1β	1.0535	1.0278–1.0799	< 0.0001	Higher MIP-1β associated with higher odds of active UC.
Model 3	GM-CSF	1.0659	1.0298–1.1033	0.0003	Conditional main effect at median MIP-1α.
Model 3	IL-5	1.4782	1.1314–1.9315	0.0042	Higher IL-5 associated with higher odds of active UC.

4.7 Spline sensitivity analysis for IL-1β in Model 3

A generalized additive model with a smooth term for IL-1β was fitted as a sensitivity analysis. Although the smooth term was statistically significant within the GAM, the effective degrees of freedom were close to linearity and the spline variant did not materially improve residual deviance compared with the linear Model 3 specification.

Table S10: Spline sensitivity analysis for IL-1β in Dataset A Model 3.

Model variant	Residual deviance	Effective df for IL-1β smooth	p-value vs. linear Model 3	Interpretation
Linear Model 3	94.90	Not applicable	Reference	Retained as the main Dataset A model.
Model 3 with spline term for IL-1β	93.454	1.515	0.1082	No clear material improvement in model fit; the linear term was retained for interpretability.

5 Dataset B: extended mediator panel

5.1 Pre-modeling decisions

Dataset B contained 67 records with an extended mediator panel. Because this dataset combined a smaller sample size with a larger number of candidate variables, it was treated as an exploratory extended-panel analysis. Stepwise regression on the initial extended variable set was not feasible due to strong collinearity among multiple mediators. Therefore, hierarchical clustering and PCA were used to summarize highly correlated variables before model-building.

Table S11: Pre-modeling decisions in Dataset B.

Variable or group	Issue encountered	Decision applied	Rationale / consequence
MCP-3	Predominant values near or below the limit of detection	Excluded from PCA construction and not retained in final Model 4	Reduced technical instability.
IL-12p40	Predominant values near or below the limit of detection and separate cluster assignment	Excluded from PCA construction and not retained in final Model 4	Prevented a low-information technical component from driving the PCA-based modelling workflow.
Highly correlated mediators	At least one Pearson correlation $r > 0.80$	Hierarchical clustering followed by PCA	Reduced collinearity and summarized correlated mediator profiles.
Variables not highly correlated with others	No PCA required	Retained as individual candidate predictors	Entered feature-reduction procedure as individual variables.
Feature selection and internal performance assessment	Feature selection performed once before CV	Post-selection 10-fold CV	Performance metrics describe internal post-selection discrimination, not validation of the whole feature-selection algorithm.

5.2 Hierarchical clustering and PCA

Hierarchical clustering was performed on variables with at least one high correlation with another variable. After exclusion of MCP-3 and IL-12p40 from PCA construction, the remaining correlated variables were subjected to PCA in standardized form. Three principal components were retained; together, they explained 83.07% of the variance among variables included in the PCA.

In the PCA tables, eigenvectors denote the raw coefficients used to construct each principal component from standardized variables, whereas component loadings denote correlations between the original standardized variables and the principal components. Component loadings were used to aid biological interpretation of PC1, which was selected into the revised Model 4. Because the sign of a principal component is arbitrary, the direction of PC1 should be interpreted together with the reported eigenvectors and loadings rather than as a directly measured biological increase or decrease in a single mediator.

Table S12: Variables subjected to PCA in Dataset B after clustering and technical exclusions.

Variable	Cluster	Included in PCA	Comment
IL-1β	1	Yes	Included in standardized PCA.
IL-4	1	Yes	Included in standardized PCA.
IL-5	1	Yes	Included in standardized PCA.
IL-8	1	Yes	Included in standardized PCA.
IL-12p70	1	Yes	Included in standardized PCA.
IL-15	1	Yes	Included in standardized PCA.
FGF2	1	Yes	Included in standardized PCA.
G-CSF	1	Yes	Included in standardized PCA.
IFNγ	1	Yes	Included in standardized PCA.
MIP-1α	1	Yes	Included in standardized PCA.
VEGF-A	1	Yes	Included in standardized PCA.
IL-3	1	Yes	Included in standardized PCA.
IFNα	1	Yes	Included in standardized PCA.
LIF	1	Yes	Included in standardized PCA.
β-NGF	1	Yes	Included in standardized PCA.
SDF-1α	1	Yes	Included in standardized PCA.
IL-12p40	2	No	Excluded due to predominant values near or below the limit of detection.
MCP-3	1	No	Excluded due to predominant values near or below the limit of detection.

Table S13: Summary of principal components derived in Dataset B.

Component	Eigenvalue	Variance explained	Cumulative variance explained	Comment
PC1	6.8331	54.59%	54.59%	Retained and selected into the revised final Model 4.
PC2	2.4147	19.29%	73.88%	Retained as a candidate component, but not selected into the revised final Model 4.
PC3	1.1510	9.19%	83.07%	Retained as a candidate component, but not selected into the revised final Model 4.
PC4	0.5329	4.26%	87.33%	Not retained.
PC5	0.4157	3.32%	90.65%	Not retained.

Table S14: PCA eigenvectors and component loadings for the retained components in Dataset B.

Variable	EV PC1	EV PC2	EV PC3	Loading PC1	Loading PC2	Loading PC3	Interpretational note for PC1
IL-4	-0.1940	0.2081	0.2238	-0.7260	0.4629	0.3437	Negative contributor to PC1.
IL-5	-0.2481	0.2274	0.1010	-0.7426	0.4046	0.1241	Negative contributor to PC1.
IL-8	-0.2363	0.1457	0.2748	-0.7368	0.2700	0.3517	Negative contributor to PC1.
IL-12p70	-0.2266	0.2692	-0.5206	-0.6253	0.4415	-0.5896	Negative contributor to PC1.
IL-15	-0.2419	0.0823	0.2195	-0.7730	0.1563	0.2879	Negative contributor to PC1.
FGF2	-0.2435	0.3166	0.1231	-0.6947	0.5371	0.1442	Negative contributor to PC1.
G-CSF	-0.2201	0.1023	0.1756	-0.7856	0.2170	0.2573	Negative contributor to PC1.
IFNγ	-0.1867	0.1702	0.1572	-0.7741	0.4195	0.2674	Negative contributor to PC1.
MIP-1α	-0.2980	0.2452	0.0286	-0.8145	0.3985	0.0321	Negative contributor to PC1.
VEGF-A	-0.2491	0.1745	-0.6461	-0.6306	0.2626	-0.6713	Negative contributor to PC1.
IL-3	-0.2867	-0.3935	0.0162	-0.7494	-0.6115	0.0173	Negative contributor to PC1.
IFNα	-0.2968	-0.3703	-0.0093	-0.7758	-0.5754	-0.0100	Negative contributor to PC1.
LIF	-0.2830	-0.2875	0.1281	-0.7398	-0.4467	0.1375	Negative contributor to PC1.
β-NGF	-0.2925	-0.3445	-0.0596	-0.7647	-0.5353	-0.0639	Negative contributor to PC1.
SDF-1α	-0.2956	-0.2494	-0.1623	-0.7728	-0.3875	-0.1741	Negative contributor to PC1.
IL-1β	-0.1354	0.1369	0.0970	-0.7168	0.4309	0.2107	Negative contributor to PC1.

5.3 Model 4 selection summary

The full stepwise trace for Dataset B was long and included repeated exclusion and re-inclusion attempts caused by the dimensionality and collinearity structure of the extended panel. To preserve interpretability, the reader-oriented Supplementary Materials summarize the final selected structure and the key pre-modeling decisions rather than reproducing the full trace. Following correction of the Dataset B modelling workflow, the revised final Model 4 retained IL-1RA, TRAIL, PC1, GRO, GM-CSF, SCF, and MIP-1β.

Table S15: Condensed summary of revised Model 4 construction in Dataset B.

Stage	Decision	Interpretation
Initial extended-panel model	Direct stepwise modelling of the full extended mediator set was attempted.	The procedure was unstable due to high collinearity and high dimensionality relative to sample size.
Collinearity handling	Variables with high correlations were clustered and summarized using PCA.	PCA reduced the dimensionality of the correlated mediator block.
Technical exclusions	MCP-3 and IL-12p40 were excluded from PCA construction due to predominant values near/below detection limits.	These markers were not used to define principal components and were not retained in revised final Model 4.
Feature-reduction stage	Stepwise selection was applied to principal components and non-PCA mediator variables.	The procedure was exploratory and post-selection uncertainty should be interpreted cautiously.
Final revised model	IL-1RA, TRAIL, PC1, GRO, GM-CSF, SCF, and MIP-1β were retained.	Model 4 represents an extended-panel exploratory discriminative model, not an externally validated classifier.

5.4 Final revised Model 4 and PC1 interpretation

Model 4 was developed in Dataset B and should be interpreted as an exploratory extended-panel model. In the stepwise output, PC1 was retained in the final model. PC1 represents an aggregate mediator profile and its association with the odds of active UC should not be interpreted as the effect of a single mediator. Because all PC1 eigenvectors and component loadings were negative in the present PCA orientation, the positive coefficient for PC1 reflects the selected direction of this latent component rather than a simple increase in all contributing mediators.

Table S16: Internal post-selection discrimination metrics for Dataset B revised Model 4.

Model	Learning AUC	Testing AUC	Sensitivity	Specificity
Model 4	0.913 ± 0.037	0.825 ± 0.056	0.640	0.952

AUC metrics were obtained after the model structure had been selected. Sensitivity and specificity were calculated in the testing subset at the probability threshold maximizing Youden's index. At this threshold, sensitivity was 0.640, specificity was 0.952, and Youden's index was 0.592. These metrics should be interpreted as internal post-selection discrimination estimates rather than as external validation of the full modelling workflow.

Table S17: Final exploratory logistic regression Model 4 in Dataset B after correction of the stepwise selection output.

Effect	Interpretation	OR	95% CI	p-value	Note
Intercept	Baseline odds of active UC for a profile with continuous non-PCA predictors at their centered reference values and PCA variables at their standardized reference.	0.1300	0.0350–0.4826	0.0023	Intercept reflects the centered/scaled model reference profile.
IL-1RA	Fold increase in the odds of active UC per one-unit increase in median-centered winsorized IL-1RA.	1.0159	1.0045–1.0274	0.0063	Higher IL-1RA associated with higher odds of active UC.
TRAIL	Fold increase in the odds of active UC per one-unit increase in median-centered winsorized TRAIL.	1.0755	1.0255–1.1278	0.0027	Higher TRAIL associated with higher odds of active UC.
PC1	Fold increase in the odds of active UC per one-unit increase in Principal Component 1.	9.0876	2.2875–36.1031	0.0017	Aggregate mediator profile; not an individual biomarker. Direction should be interpreted with the PC1 eigenvectors and loadings.
GRO	Fold increase in the odds of active UC per one-unit increase in median-centered winsorized GRO.	1.0314	1.0117–1.0515	0.0017	Higher GRO associated with higher odds of active UC.
GM-CSF	Fold increase in the odds of active UC per one-unit increase in median-centered winsorized GM-CSF.	1.0988	1.0329–1.1689	0.0028	Higher GM-CSF associated with higher odds of active UC.
SCF	Fold increase in the odds of active UC per one-unit increase in median-centered winsorized SCF.	1.0340	1.0007–1.0685	0.0454	Higher SCF associated with higher odds of active UC; borderline lower confidence bound.
MIP-1β	Fold increase in the odds of active UC per one-unit increase in median-centered winsorized MIP-1β.	1.0512	1.0119–1.0919	0.0101	Higher MIP-1β associated with higher odds of active UC.

Table S18: Approximate contribution of PC1 variables to the direction of association in revised Model 4.

Variable	Approximate interpretation	EV for PC1	Approximate OR	Interpretational note
MIP-1α	Per one SD increase in MIP-1α along the PC1 eigenvector direction	-0.2980	0.5181	Strong negative contributor to the PC1 direction.
IFNα	Per one SD increase in IFNα along the PC1 eigenvector direction	-0.2968	0.5195	Strong negative contributor to the PC1 direction.
SDF-1α	Per one SD increase in SDF-1α along the PC1 eigenvector direction	-0.2956	0.5208	Strong negative contributor to the PC1 direction.
β-NGF	Per one SD increase in β-NGF along the PC1 eigenvector direction	-0.2925	0.5243	Strong negative contributor to the PC1 direction.
IL-3	Per one SD increase in IL-3 along the PC1 eigenvector direction	-0.2867	0.5312	Strong negative contributor to the PC1 direction.
LIF	Per one SD increase in LIF along the PC1 eigenvector direction	-0.2830	0.5355	Strong negative contributor to the PC1 direction.
VEGF-A	Per one SD increase in VEGF-A along the PC1 eigenvector direction	-0.2491	0.5771	Negative contributor to the PC1 direction.
IL-5	Per one SD increase in IL-5 along the PC1 eigenvector direction	-0.2481	0.5783	Negative contributor to the PC1 direction.
FGF2	Per one SD increase in FGF2 along the PC1 eigenvector direction	-0.2435	0.5843	Negative contributor to the PC1 direction.
IL-15	Per one SD increase in IL-15 along the PC1 eigenvector direction	-0.2419	0.5864	Negative contributor to the PC1 direction.
IL-8	Per one SD increase in IL-8 along the PC1 eigenvector direction	-0.2363	0.5936	Negative contributor to the PC1 direction.
IL-12p70	Per one SD increase in IL-12p70 along the PC1 eigenvector direction	-0.2266	0.6065	Negative contributor to the PC1 direction.
G-CSF	Per one SD increase in G-CSF along the PC1 eigenvector direction	-0.2201	0.6152	Negative contributor to the PC1 direction.
IL-4	Per one SD increase in IL-4 along the PC1 eigenvector direction	-0.1940	0.6517	Negative contributor to the PC1 direction.
IFNγ	Per one SD increase in IFNγ along the PC1 eigenvector direction	-0.1867	0.6622	Negative contributor to the PC1 direction.
IL-1β	Per one SD increase in IL-1β along the PC1 eigenvector direction	-0.1354	0.7417	Negative contributor to the PC1 direction.

Note. For all rows in Table S18, the PC1 model coefficient used for the approximate calculations was $\beta_{\text{PC1}} = 2.2069$. The approximations in Table S18 are intended to aid interpretation of the PC1 direction. They should not be interpreted as separate single-marker regression effects, because the model included PC1 as a latent component rather than the individual standardized mediators. Because all PC1 eigenvectors were negative in the present PCA orientation, the apparent direction of the approximate ORs depends on the arbitrary sign convention of the principal component.

6 Overall interpretation and limitations of the exploratory modelling workflow

The models presented in these Supplementary Materials should be interpreted as exploratory discriminative summaries of circulating immunomodulator profiles. They do not establish causal effects of individual mediators and should not be interpreted as externally validated diagnostic tools.

The feature-reduction procedures were used to obtain parsimonious models from high-dimensional mediator data relative to the available sample size. Therefore, the selected variables and principal components should be viewed as candidate signals requiring independent validation. The p-values reported for final model terms are post-selection p-values and should be interpreted as descriptive evidence within the fitted models rather than as confirmatory evidence for independent biomarkers.

The internal cross-validation metrics were calculated after the final model structures had been selected. Consequently, these metrics describe post-selection internal discrimination of the selected models. They do not estimate the full out-of-sample performance of the complete model-building process, including feature selection, and they do not replace validation in an independent cohort.

The extended-panel analysis in Dataset B should be considered particularly hypothesis-generating because it combined a smaller sample size with a larger number of candidate mediators and dimension-reduction procedures. In the revised Model 4, PC1 should be interpreted as an aggregate immune mediator profile, not as a directly measured biological marker.

Taken together, the modelling results suggest that circulating immunomodulator profiles contain a multivariable signal distinguishing active UC from active CD in this cohort. However, the selected models should be treated as exploratory summaries and candidates for future validation rather than as final diagnostic classifiers.

7 Extended audit trail note

The original spreadsheet outputs contain full stepwise traces, including candidate terms tested but not retained at each step. These traces are lengthy, particularly for Dataset B. To avoid obscuring the main analytical narrative, the present Supplementary Materials report condensed model-changing decisions, final model terms, PCA details, model comparison metrics, and explicit interpretation limits.
